# A Gridded Inventory of Annual 2012–2018 U.S. Anthropogenic Methane Emissions

**DOI:** 10.1021/acs.est.3c05138

**Published:** 2023-10-19

**Authors:** Joannes D. Maasakkers, Erin E. McDuffie, Melissa P. Sulprizio, Candice Chen, Maggie Schultz, Lily Brunelle, Ryan Thrush, John Steller, Christopher Sherry, Daniel J. Jacob, Seongeun Jeong, Bill Irving, Melissa Weitz

**Affiliations:** † SRON Netherlands Institute for Space Research, Leiden 3584 CA, Netherlands; ‡ Climate Change Division, Environmental Protection Agency, Washington, District of Columbia 20004, United States; § School of Engineering and Applied Sciences, Harvard University, Cambridge, Massachusetts 02138, United States; ∥ Lawrence Berkeley National Laboratory, Berkeley, California 94720, United States

**Keywords:** greenhouse gas, UNFCCC, agriculture, petroleum, natural gas, coal, waste

## Abstract

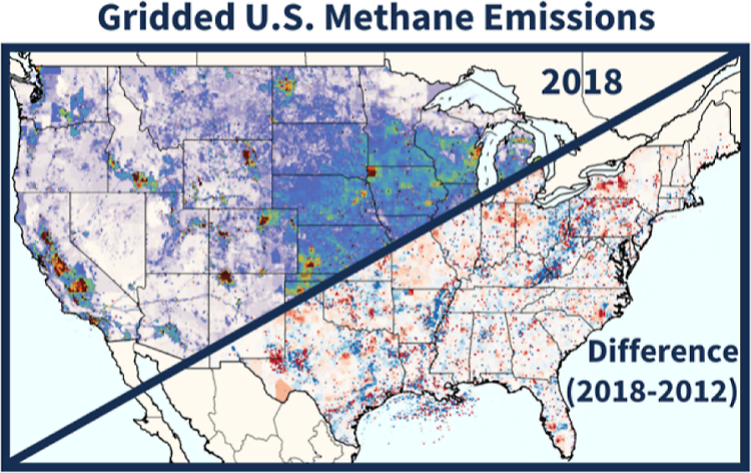

Nationally reported greenhouse gas inventories are a core component of the Paris Agreement’s transparency framework. Comparisons with emission estimates derived from atmospheric observations help identify improvements to reduce uncertainties and increase the confidence in reported values. To facilitate comparisons over the contiguous United States, we present a 0.1° × 0.1° gridded inventory of annual 2012–2018 anthropogenic methane emissions, allocated to 26 individual source categories, with scale-dependent error estimates. Our inventory is consistent with the U.S. Environmental Protection Agency (EPA) Inventory of U.S. Greenhouse Gas Emissions and Sinks (GHGI), submitted to the United Nations in 2020. Total emissions and patterns (spatial/temporal) reflect the activity and emission factor data underlying the GHGI, including many updates relative to a previous gridded version of the GHGI that has been extensively compared with observations. These underlying data are not generally available in global gridded inventories, and comparison to EDGAR version 6 shows large spatial differences, particularly for the oil and gas sectors. We also find strong regional variability across all sources in annual 2012–2018 spatial trends, highlighting the importance of understanding regional- and facility-level activities. Our inventory represents the first time series of gridded GHGI methane emissions and enables robust comparisons of emissions and their trends with atmospheric observations.

## Abstract 2

We present time series of gridded (∼10 × 10 km^2^) anthropogenic methane emissions, consistent with the USEPA greenhouse gas inventory, that facilitates observation-based evaluations.

## 1 Introduction

Reductions in methane emissions are an important factor in reaching collective climate goals, such as limiting global mean warming to below 2 °C.^1,2^ Inventories of greenhouse gas (GHG) emissions, including methane, are key to supporting and tracking these goals by, for example, supporting the development and tracking of domestic mitigation policies,^3^ Nationally Determined Contributions, and informing the Paris Agreement’s Global Stocktake.^1^ Under the 1992 United Nations Framework Convention on Climate Change (UNFCCC),^4^ Parties are required to report inventories of anthropogenic GHG emissions and sinks using internationally agreed-upon methodological guidance from the Intergovernmental Panel on Climate Change (IPCC).^5,6^ The quality of GHG inventory estimates is dependent on the underlying emission mechanisms and the robustness of methods and data used. In some cases, particularly for some methane sources, limited or uncertain underlying data can result in large uncertainties.^6^ As described in the 2019 Refinements to the IPCC GHG Guidelines,^6^ estimates can be compared to emissions derived from independent atmospheric observations, as a part of a broader strategy to evaluate and improve inventories. Comparisons with observations can provide information to identify key areas for refinement, particularly for methane, as emissions are largely from fugitive and biological sources, which can be more challenging to quantify than other GHG sources. Here, we present the first time series of gridded U.S. anthropogenic methane emissions, consistent with national U.S. UNFCCC reporting, to facilitate these observation-based comparisons.

In the United States, national time series of source-specific anthropogenic GHG emissions are reported annually in the Inventory of U.S. Greenhouse Gas Emissions and Sinks (GHGI, Table 1), produced by the U.S. Environmental Protection Agency (EPA). National U.S. methane emissions in 2018 (as reported in 2020) were estimated at 25.4 Tg (95% confidence interval: +5%, −14%),^7^ which accounts for ∼7% of global 2018 anthropogenic methane emissions.^8^ However, several studies using ground, aircraft, and satellite observations of atmospheric methane have indicated that there may be large uncertainties across national estimates.^9–14^ For example, previous comparisons in the United States have suggested higher methane emissions from oil and gas production than in the GHGI,^9,15–18^ especially over the Permian oil production area,^19^ and have pointed out large regional contributions (∼up to 40%^20^) from “super-emitting” facilities (>10 kg h^–1^).^20,21^ In other production areas, studies have found better agreement with the GHGI.^16,22,23^ Studies of urban areas have also pointed out underestimation of urban methane emissions in the GHGI,^24–27^ in part associated with landfills and natural gas distribution and end use.

**Table 1:** Anthropogenic Methane Emission (kt yr^–1^) and Uncertainties for 2012 and 2018 from the 2020 U.S. GHGI,^7^ in Order of Decreasing Emissions

source (CRF^a^ category)	2020 U.S. GHGI methane emissions (kt)^b^			
	2012 emissions	2018 emissions	95% confidence interval^c^	percent change (%)^d^
**total (without LULUCF)**^e^	**25,873**	**25,378**	**–5%, +14%**	**–1.9**
**agriculture**	**9568**	**10,119**		**+5.8**
enteric fermentation (3A)	6670	7103	–11%, +18%	+6.5
manure management (3B)^f^	2278	2467	–18%, +20%	+8.3
rice cultivation (3C)^f^	606	533	–31%, +62%	–12.0
field burning of agricultural residues (3F)^f^	14	16	–16%, +16%	+14.3
**natural gas systems (1B2b)**	**5656**	**5598**	**–15%, +14%**^g^	**–1.0**
production^f^	3490	3238		–7.2
transmission& storage	1166	1355		+16.2
processing	400	488		+22.0
distribution	500	473		–5.4
exploration^f^	100	44		–56.0
**waste**	**5322**	**5089**		**–4.4**
municipal solid waste (MSW) landfills (5A1)	4070	3823	–25%, +25%	–6.1
industrial landfills (5A1)	593	599	–31%, +25%	+1.0
domestic wastewater treatment and discharge (5D)	360	334	–28%, +22%	–7.2
industrial wastewater treatment and discharge (5D)	222	235	–48%, +50%	+5.9
composting (5B1)	77	98	–50%, +50%	+27.3
**coal mines (1B1a)**	**2907**	**2356**		**–19.0**
underground coal mining	2159	1768	–17%, +12%	–18.1
surface coal mining	499	341	–17%, +12%	–31.7
abandoned underground coal mines	249	247	–20%, +15%	–0.8
**petroleum systems (1B2a)**^f^	**1631**	**1449**	**–31%, +34%**	**–11.2**
production	1289	1395		+8.2
refining	30	31		+3.3
exploration	306	15		–95.1
transport	6	8		+33.3
**other**	**790**	**762**		**–3.5**
stationary combustion (1A)^f^	304	344	–35%, +130%	+13.2
abandoned oil and gas wells (1B2a and 1B2b)	282	281	–83%, +219%	–0.4
mobile combustion (1A)	200	124	–8%, +27%	–38.0
petrochemical production (2B8)	3	12	–57%, +46%	+300
ferroalloy production (2C2)	1	1	–12%, +12%	0

a Categories reported in UNFCCC Common Reporting Format tables.

b The GHGI is updated on an annual basis; values shown here are from the 2020 published GHGI. The express data set is consistent with national emissions from the 2022-published GHGI.

c 95% confidence interval for 2018 from the 2020 GHGI.

d Calculated as 100 × (2018 emissions – 2012 emissions)/2012 emissions.

e LULUCF: land use, land use change, and forestry. In the 2020 GHGI, these categories contribute to an additional ∼600 kt of methane emissions in 2018. These sources are not included in gridded emission estimates due to limited information. For inverse modeling applications, global emission databases can be used for these sources.

f Source sectors that include annual gridded emissions and monthly scale factors.

g Error estimates are not reported in the GHGI for segment-level emissions.

Inverse analyses of atmospheric observations require a gridded emission inventory as a prior estimate.^28^ This inventory then serves as the basis for interpreting the inversion results. Most of the above studies used the gridded U.S. methane emissions data set as a prior estimate from Maasakkers et al. (2016),^29^ which represents emissions in a single year (2012) and is based on the 2016-reported GHGI.^30^ Other studies have relied on the global gridded inventory from the Emissions Database for Global Atmospheric Research (EDGAR),^31,32^ which shows large inconsistencies with the GHGI.^29^ While these gridded inventories have enabled comparisons with observations, they tend to be inconsistent with current national emission estimates and trends. The U.S. GHGI is updated annually with new methods and/or data to improve inventory quality, completeness, and consistency and to reduce uncertainties. This has led, for example, to recent increases in reported methane emissions from oil and gas production, in part due to the inclusion of several large well blowout events (>4 kt) that were added based on quantifications of satellite observations,^33,34^ as well as emissions (leakage) from abandoned oil and gas wells and “end-use” sources downstream of natural gas distribution meters. The use of inconsistent gridded products as prior estimates in inversions can lead to biases^35^ and misinterpretation of the observation-based results.

We present spatially disaggregated 0.1° × 0.1° annual emission maps and monthly scaling factors of U.S. anthropogenic methane emissions for 2012–2018, consistent with the 2020 GHGI.^7^ This allows for the evaluation of GHGI spatial trends over time as a function of 26 individual source categories. In this work, we better align our spatial disaggregation patterns with data sets underlying the GHGI compared to previous U.S. gridded estimates.^29^ We also capture recent GHGI methodological improvements and recently added emission sources and changes in spatial patterns over time.^36^ Furthermore, by recognizing the need for contemporary gridded estimates to compare to the increasing volume of atmospheric observations, we also present an extended “express” data set that provides annual gridded emission estimates for 2012–2020, consistent with national emissions from the GHGI, published in 2022,^33^ but based on the spatial disaggregation developed here for the 2020 GHGI.^7^

## 2 Methods

**Figure 1:**
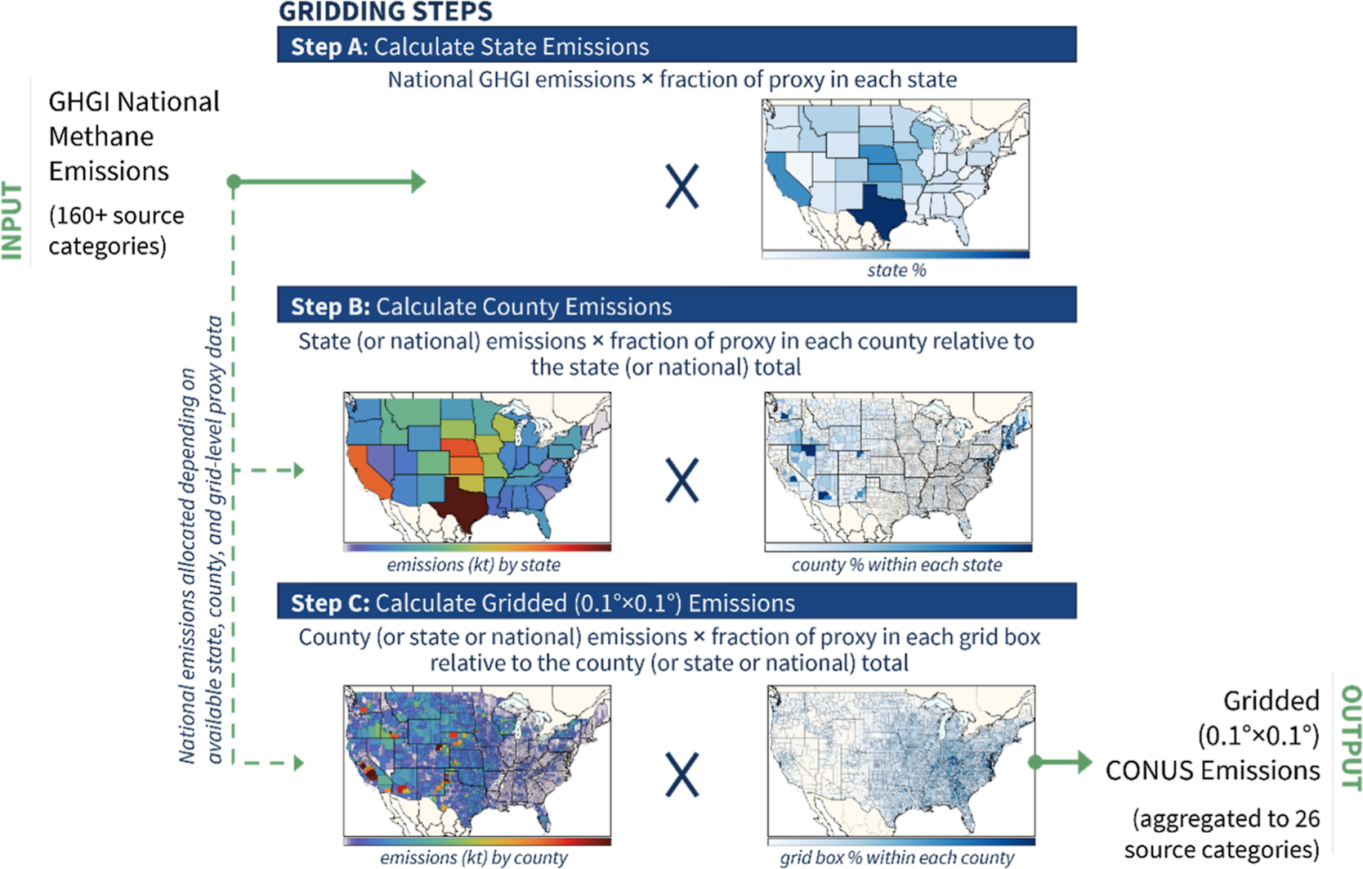
Schematic of the gridding methodology showing the gridding of enteric fermentation emissions as an example. (Step A) State emissions are allocated based on national emissions multiplied by the fraction of proxy data in each state. (Step B) County emissions are allocated based on state-level emissions multiplied by the fraction of proxy data in each county. (Step C) Gridded emissions are allocated based on county-level emissions multiplied by the fraction of proxy data within each grid cell.

National annual 2012–2018 GHGI methane emissions from over 160 individual sources are allocated to a 0.1° × 0.1° (∼10 × 10 km) grid using a series of spatial and temporal proxy data sets at the state, county, and grid levels (Figure 1). We use proxy data to best align with the available activity and reported emissions data underlying the 2020 GHGI. Where possible, the proxy data are the same as those used to develop the GHGI. For example, we use the same oil and gas well data as is used in the GHGI, as well as the same facility-level data from EPA’s Greenhouse Gas Reporting Program (GHGRP),^37^ which is used for the compilation of emissions for many GHGI sources. The GHGRP collects reported data (starting in 2011) from facilities that annually emit above the reporting threshold of 25 Mmt CO_2_ equivalent. For other sources with more limited underlying spatial or temporal information, emissions are allocated using proxies as determined by expert elicitation. As shown in Figure 1, to account for differences in available source-specific information, emissions from each source category are allocated in a stepwise proportional approach (steps A–C in Figure 1 and Table S1). For example, the locations of household residential wood combustion emissions (source category 1A) are not precisely known. Therefore, national emissions are first distributed across each state and county using fractional amounts of residential wood consumption (steps A and B) and are then distributed to the grid level based on population (step C). As another example, in cases where facility-level information is known, national emissions are allocated directly to each grid cell using proportional facility-level data (step C only). Final gridded emissions from the ∼160 individual GHGI sources are then aggregated into the final 26 source categories listed in Table 1. Consistent with Maasakkers et al. (2016),^29^ our geographic domain is limited to the contiguous U.S. (CONUS) (Table S1). Monthly varying emission scale factors are included for sources with large temporal variability (Table 1). The following sections summarize the approach and data used for each source category. We also describe our error characterization approach, as well as an annual 2012–2020 “express” extension, which is consistent with national emissions from the 2022 GHGI^33^ but follows the spatial patterns from our main (2012–2018) product. As described in detail below, the express data set was developed to provide the best representation of a more recent GHGI before a full gridded update could be completed.

### 2.1 Agriculture

#### 2.1.1 Livestock—Manure Management and Enteric Fermentation

We start with annual state-level GHGI livestock methane emissions, developed as a function of over 10 individual animal types. We spatially distribute these emissions based on relative county-level animal counts from (interpolation of) the 2012 and 2017 U.S. Department of Agriculture (USDA) Census of Agriculture.^38^ In the absence of a national farm-level data set, county-level emissions are then allocated to the 0.1° × 0.1° CONUS grid using gridded livestock occurrence probability maps, based on land types, for nine aggregated animal-type groups from the USDA.^39,40^ On average, a county covers ∼25 0.1 × 0.1° grid cells. In addition, emissions from manure management are also expected to have a strong seasonal dependence, largely due to the temperature-dependent biological availability of volatile solids in manure systems.^5,29,41^ To capture these variations, we generate monthly gridded scale factors for this source for each year using monthly state-level methane emissions data from the GHGI, as a function of animal type and waste management system. In contrast, we do not estimate monthly seasonal scale factors for enteric fermentation as methane emissions from this source are less directly temperature-dependent and are not calculated as such in the GHGI.

#### 2.1.2 Rice Cultivation

Annual GHGI state-level estimates for the 13 rice-producing states are distributed to counties based on the acres of rice harvested, derived from (interpolation of) the 2012 and 2017 USDA Census.^38^ Emissions are gridded using annual 30 m resolution rice crop maps from the USDA Cropland Data Layer (CDL) Product.^42^ We allocate emissions to months by applying normalized mean 2001–2010 heterotrophic respiration rates from the Carbon Data-Model Framework (CARDAMOM).^43^

#### 2.1.3 Field Burning of Agricultural Residues

Annual state GHGI emissions as a function of 21 crop types (90% of emissions are associated with corn, cotton, rice, soybeans, and wheat) are gridded using a monthly climatology of agricultural fire emissions for six crop categories (corn, cotton, rice, soybeans, wheat, and other).^44^

### 2.2 Energy—Natural Gas Systems

Emissions from Natural Gas Systems arise from the exploration, production, processing, transmission, storage, and distribution of natural gas. The GHGI estimates these emissions using activity and emission factor data for over 100 individual activities and equipment types (e.g., well completions, distribution pipelines, and so forth). As described below, we use spatial information from these data sets, where available, and otherwise allocate emissions based on sources with common features. We provide monthly emission scale factors for the production and exploration segments and annual emissions for all other segments.

#### 2.2.1 Production and Exploration

We largely grid emissions using the Enverus (formerly DrillingInfo) well-level data set^45^ that was used to develop the 2020 GHGI. This data set includes annual well-specific information such as locations, gas production volumes, and completion dates. Wells are classified as gas wells if their gas/oil production ratio is over 100 mcf bbl^–1^. Enverus data are not available for Indiana and Illinois, where we instead grid emissions using 4 × 4 km maps of annual well-level data developed as part of the National Emissions Inventory (NEI).^46^ For sources related to condensate, annual condensate production data from the Energy Information Administration (EIA)^47^ are used to allocate emissions to the state level before gridding using Enverus gas well locations. For offshore platforms in federal waters in the Gulf of Mexico, emissions are gridded using relative platform-specific emissions from the (interpolation of) 2011, 2014, and 2017 Bureau of Ocean Energy Management (BOEM) Gulfwide Emission Inventories.^48^ We grid national emissions from gathering and boosting (G&B) by uniformly allocating them across gathering compressor station locations or miles of gathering pipelines from the 2021 Enverus Midstream infrastructure data set.^49^ This is an improvement over Maasakkers et al. (2016)^29^ who spatially allocated G&B emissions using the same spatial pattern as other production segment emissions. We estimate monthly emission scale factors for all sources based on monthly well/platform-level gas production volumes but assume no intramonthly variability for gathering and boosting.^45^

#### 2.2.2 Processing

National emissions from the processing segment are spatially allocated based on estimated relative plant-specific methane emissions. We estimate plant specific emissions using annual data from the U.S. GHGRP^37^ (∼40% of plants) and the Enverus Midstream infrastructure data set^49^ using a combination of reported emissions and facility-level emission-to-throughput ratios.

#### 2.2.3 Transmission and Storage

Emissions at Liquified Natural Gas (LNG) Import and Export terminals are uniformly allocated to locations of operational terminals that are listed each year in the Department of Energy LNG Annual Reports.^50^ Similarly, emissions at LNG storage stations are allocated based on annual station-specific storage capacities from the Pipeline and Hazardous Materials Safety Administration (PHMSA).^51^ The PHMSA data set only includes zip codes, and therefore specific locations are derived by matching PHMSA station names to those in the Enverus Midstream data set^49^ (∼15% of stations) and peak shaving facilities from the FracTracker Alliance.^52^ National emissions from underground storage wells are gridded based on annual storage capacities at storage field locations from EIA,^53^ supplemented with methane emissions from the Aliso Canyon blowout event in 2015 and 2016, as included in the GHGI.^54,55^ For transmission compressor stations, we grid national GHGI emissions based on relative annual GHGRP^37^ emissions for ∼570 reporting stations and estimate relative emissions from all other stations (∼1600) using emissions and fuel use data from the GHGRP and Enverus infrastructure data sets.^49^ Similarly, for storage compressor stations, we use annual relative emissions from ∼50 GHGRP-reporting stations and estimate relative emissions at the remaining (∼300) nonreporting stations using the ratio between GHGRP-reported emissions and field-specific gas storage capacities.^53^ Last, leaks from transmission pipelines and meter and regulating stations are gridded based on locations and miles of transmission pipelines from Enverus,^49^ while annual emissions from farm taps are allocated to grid cells where transmission pipelines intersect agricultural land.^42^

#### 2.2.4 Distribution

Emissions from pipeline and service leaks are allocated to the state level using annual state-specific miles of distribution pipelines, as a function of pipeline material (cast iron, unprotected/protected steel, and plastic) and the number of service stations.^56^ Metering and regulating emissions at city gates are allocated using annual state-level counts of above- and below-grade service stations from the GHGRP,^37^ while commercial, residential, and industrial customer meter emissions are allocated using annual state-level counts of consumers from the EIA.^57^ State-level emissions for all distribution sources are then gridded using population.^58^

### 2.3 Waste

#### 2.3.1 Landfills

Emissions from municipal solid waste (MSW) landfills are gridded using annual relative MSW landfill emissions reported to the GHGRP.^37^ Emissions from additional nonreporting MSW landfills (9–11% of emissions) are distributed using “waste in place” data and landfill locations underlying the GHGI.^59^ Industrial landfill emissions associated with pulp and paper and food and beverage manufacturing are allocated to the CONUS grid using a combination of annual GHGRP data,^37^ 2016 pulp and paper plant locations,^60^ amounts of excess food waste,^61^ and facility locations from the U.S. EPA Facility Registry Service (FRS).^62^

#### 2.3.2 Wastewater Treatment and Discharge

The GHGI considers treatment of domestic wastewater through septic systems (∼65% of emissions) and three types of centralized publicly owned treatment works (POTWs): anaerobic, aerobic, and anaerobic digestors. We grid national emissions from septic systems using population.^58^ Emissions from POTWs are gridded using facility-level locations, annual wastewater flow rates, and flow capacities from EPA’s Enforcement and Compliance History Online (ECHO) data set.^63^ Each facility is classified as aerobic, anaerobic, or having an anaerobic digestor using the latest available facility treatment-type data from the 2004 Clean Watershed Needs Survey.^64^ The GHGI includes industrial wastewater emissions from six distinct activities: pulp and paper, red meat and poultry, fruit and vegetables, ethanol production, petroleum refining, and breweries. We grid national industry-specific emissions using annual GHGRP emissions^37^ and annual locations and wastewater flow rates for industry-specific, non-POTW facilities from EPA’s ECHO database.^63^

#### 2.3.3 Composting

We grid GHGI state-level emissions using facility location information from the EPA,^61^ U.S. Compost Council,^65^ BioCycle composter database,^66^ and the FRS.^62^ For states with fewer than two facilities, state-level emissions are gridded based on population.^58^

### 2.4 Energy—Coal Mines

#### 2.4.1 Active Coal Mining

For active underground mines, annual net state-level GHGI emissions are gridded based on annual mine-specific relative emissions from the GHGRP,^37^ as well as emissions calculated from annual mine-specific coal production from the EIA,^67^ weighted by basin-level in situ methane coal content in states with multiple basins.^7^ Active surface mines do not report to the GHGRP, and therefore annual net GHGI state-level emissions (surface mining + postmining activities) are gridded using mine-specific coal production from the EIA,^67^ also weighted by methane content for states with multiple coal basins. All mine locations are from the Mine Safety and Health Administration (MSHA),^68^ which is an improvement over Maasakkers et al. (2016).^29^

#### 2.4.2 Abandoned Underground Coal Mines

To estimate mine-specific emissions, we use emission decay curves from the GHGI, which are based on the time since mine closure, mine status (venting, sealed, and flooded), basin, and the emission rate when the mine was last active.^7^ If the status of a mine is unknown, we calculate emissions weighted based on the relative percentages of sealed, flooded, and vented mines within the same basin. Emissions are proportionally reduced if the mine was closed during the considered year. Mine locations are taken from the MSHA database.^68^ For abandoned mines without precise MSHA locations (∼20% or 100 mines), emissions are spread uniformly across the reported county.^68^

### 2.5 Energy—Petroleum Systems

Methane emissions from Petroleum Systems include those from onshore and offshore exploration and production (95% of emissions), transport, and refining of crude oil. Similar to Natural Gas Systems, GHGI emissions are calculated as the aggregate of activity and emission factor data associated with over 80 individual sources (e.g., well completion, major and minor offshore complexes, and so forth). We estimate monthly emission scale factors for sources based on monthly well/platform-level oil production volumes but assume no intramonthly variability for refining.

#### 2.5.1 Exploration and Production

We use GHGI-consistent Enverus well-level data^45^ to spatially allocate many of the individual emission sources. These data include annual oil well locations, well classifications (conventional vs hydraulically fractured), monthly production volumes (for onshore and offshore wells in state waters), and well drilling/completion status. Wells are classified as oil wells if their gas to oil production ratio is under 100 mcf bbl^–1^. Emissions for Indiana and Illinois are gridded based on annual 4 × 4 km NEI well-level maps,^46^ similar to Natural Gas Systems. Emissions from offshore platforms in federal waters in the Gulf of Mexico are allocated using relative platform-specific emissions from the (interpolation of) 2011, 2014, and 2017 BOEM Gulfwide Emission Inventories.^48^ Emissions in Pacific federal waters are allocated based on annual platform production volumes.^69^

#### 2.5.2 Transport and Refining

National crude oil transport emissions associated with tanks, pipeline pigging, pump stations, and floating roof tanks, as well as all refining emissions, are gridded based on annual relative oil refinery methane emissions from the GHGRP.^37^ Transport segment emissions from pump station maintenance, truck and rail, and marine loading are gridded using annual relative on- and offshore production volumes and well/platform locations from Enverus.^45^

### 2.6 Other

#### 2.6.1 Stationary Combustion

GHGI emissions from stationary combustion are calculated as a function of fuel type (coal, fuel oil, natural gas, and wood) and sector (electric power generation, industrial, commercial/institutional, and residential). We grid emissions from the electric power sector using annual fuel-specific heat input data from the U.S. EPA Acid Rain Program (ARP).^70^ We allocate industrial sector emissions to each state using annual fuel-specific energy consumption statistics from the EIA State Energy Data System (SEDS)^71^ and then grid estimates using annual plant-specific emissions from the GHGRP.^37^ Similarly, state-level commercial/institutional and residential emissions are allocated using annual fuel-specific SEDS data but are then gridded based on population.^58^ Wood combustion makes up 80% of residential and 40% of all stationary combustion methane emissions. Therefore, we add a county-level allocation step for this source using county-level residential wood consumption estimates from the latest NEI.^72^

#### 2.6.2 Abandoned Oil and Gas Wells

Emissions from abandoned oil and gas wells were added to the GHGI after publication of Maasakkers et al. (2016)^29^ and include emissions from multiple types of orphaned and nonproducing wells. We use annual GHGI state-level counts of abandoned oil and gas wells, the well region, and the well plugging status (plugged or not plugged) to allocate emissions to each state. These counts are derived from annual Enverus data and historical state-level data from the U.S. Geological Survey.^36^ State-level emissions are then gridded as a function of well type (oil or gas) using post-1975 abandoned well counts from the Enverus data set, assuming that wells abandoned prior have the same spatial pattern.

#### 2.6.3 Mobile Combustion

We calculate annual state-level emissions for on-road vehicles using annual vehicle miles traveled as a function of six vehicle types and two functional highway systems (rural and urban) from the U.S. Department of Transportation (DOT).^73,74^ State emissions are then gridded based on miles of roadways from annual maps of urban and rural roadways, derived from U.S. Census^75^ and DOT data.^76^ National emissions from other mobile sources: ships, trains, aircraft, farm and construction equipment, and “other” are gridded using annual maps of navigable waterways,^77^ U.S. Census rails data,^75^ USDA total crop areas,^42^ MSHA coal mine counts,^68^ and population,^58^ respectively.

#### 2.6.4 Ferroalloy and Petrochemical Production

Emissions from both industrial sources are gridded using annual facility-level GHGRP methane emissions and locations.^37^

### 2.7 Uncertainty Estimate

We provide resolution-dependent and source-specific uncertainty estimates to facilitate comparison of our gridded emissions to atmospheric observations, for example, through inverse analysis. Maasakkers et al. (2016)^29^ introduced a scale-dependent error variance on the emission magnitude, combined with a displacement error to characterize uncertainty in the precise location of emissions. Here, we remove the displacement error as its values are artificially small in scale because of the assumption of isotropic error in a statistical ensemble, and we find that they alias some of the error in emission magnitude. We estimate error variances by comparing our source-specific 2012 gridded estimates to those from an external detailed bottom-up inventory for the Barnett region in Texas that was compiled in 2015 and matches atmospheric measurements.^78,79^ We express the uncertainties for each source sector (σ) as a function of resolution (τ)
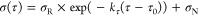
1where σ_R_ is the maximum resolution-dependent error at the native resolution of the inventory (τ_0_ = 0.1°), *k*_τ_ captures how that error decreases with spatial aggregation, and σ_N_ is the source’s national error from the GHGI. The first two parameters are optimized by minimizing the (squared summed) difference between the estimated uncertainty based on eq 1 and the absolute difference between our gridded and the Barnett inventory (taken as the best available representation of true emissions) at different resolutions.

### 2.8 Express Extension of Gridded Methane Emissions to the 2022 GHGI

Due to the significant additional analytical work required, the development of gridded emission maps can lag the publication of annually updated national inventories. Since the publication of the 2020 GHGI, the EPA has made several improvements to the GHGI, impacting methane emission estimates across the (extended) time series. To incorporate more recent inventory improvements and enable comparisons to more recent methane observations, we also report an “express” version of the gridded data set that extends the same gridding methodology described above to provide an approximate spatial allocation of annual 2012–2020 methane emissions from the more recent 2022 GHGI.^33^ This express data set is developed using the same source-specific emission patterns discussed in the previous sections (held constant after 2018 and not incorporating state-level estimates from the 2022 GHGI). Therefore, for 2012–2018, the magnitude of CONUS emissions in the express data set reflects changes in national emissions resulting from GHGI updates since the 2020 Report. The relative spatial patterns of emissions in these years are unchanged. For years after 2019, the emission maps in the express data set represent approximate spatial patterns in emissions and do not capture temporal changes in the underlying spatial proxy data since 2018.

One new non-LULUCF methane emission source was added to the 2022 GHGI for postmeter emissions and is also included in the express data set. This source captures emissions downstream of natural gas distribution meters (i.e., “Post Meter”) and accounted for ∼2% of national methane emissions in 2020 (as reported in 2022).^33^ To include this source in the express data set, we spatially allocate emissions from residential and commercial postmeter activities to each state using annual EIA counts of residential and commercial customers,^57^ which are then gridded based on population.^58^ Industrial postmeter emissions are allocated using annual state-level EIA SEDS data^71^ and then gridded using GHGRP emissions.^37^ Additional postmeter emissions associated with electricity-generating units are directly gridded using annual EPA ARP data,^70^ while natural gas vehicle emissions are allocated using state-level GHGI natural gas vehicle counts^80^ and gridded based on population.^58^

Monthly seasonal variations were not estimated directly for the express extension data set. For years 2012–2018, we recommend using the relative seasonal scaling factors from the main gridded data set to estimate monthly emissions. For years after 2018, we recommend using the seasonal monthly scaling factors for manure management, rice cultivation, and field burning of agricultural residues only. For other sources, monthly variability is too year-specific and should not be extrapolated to the express extension data for years after 2018. While this express data set enables more direct comparisons with recent observations and better reflects the latest national GHGI emission estimates, the 2012–2018 gridded emissions are the most accurate representation of the geographic distribution of methane emissions from the 2020 GHGI Report and are therefore the focus of the following Results and Discussion section.

## 3 Results and Discussion

**Figure 2:**
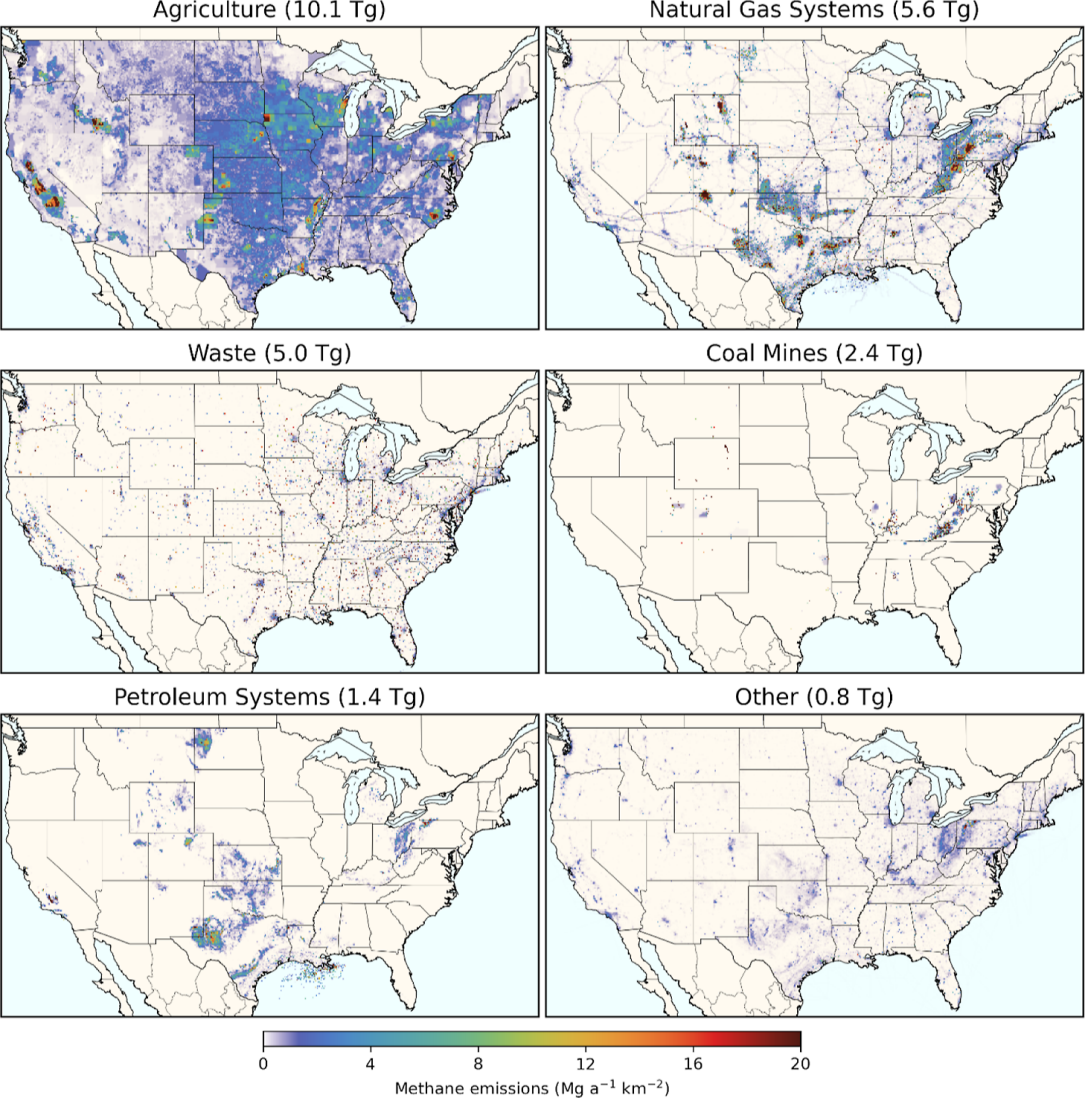
Gridded 2018 CONUS methane emission fluxes, split among six aggregate source groups. CONUS totals for 2018 are given in subplot titles.

**Figure 3:**
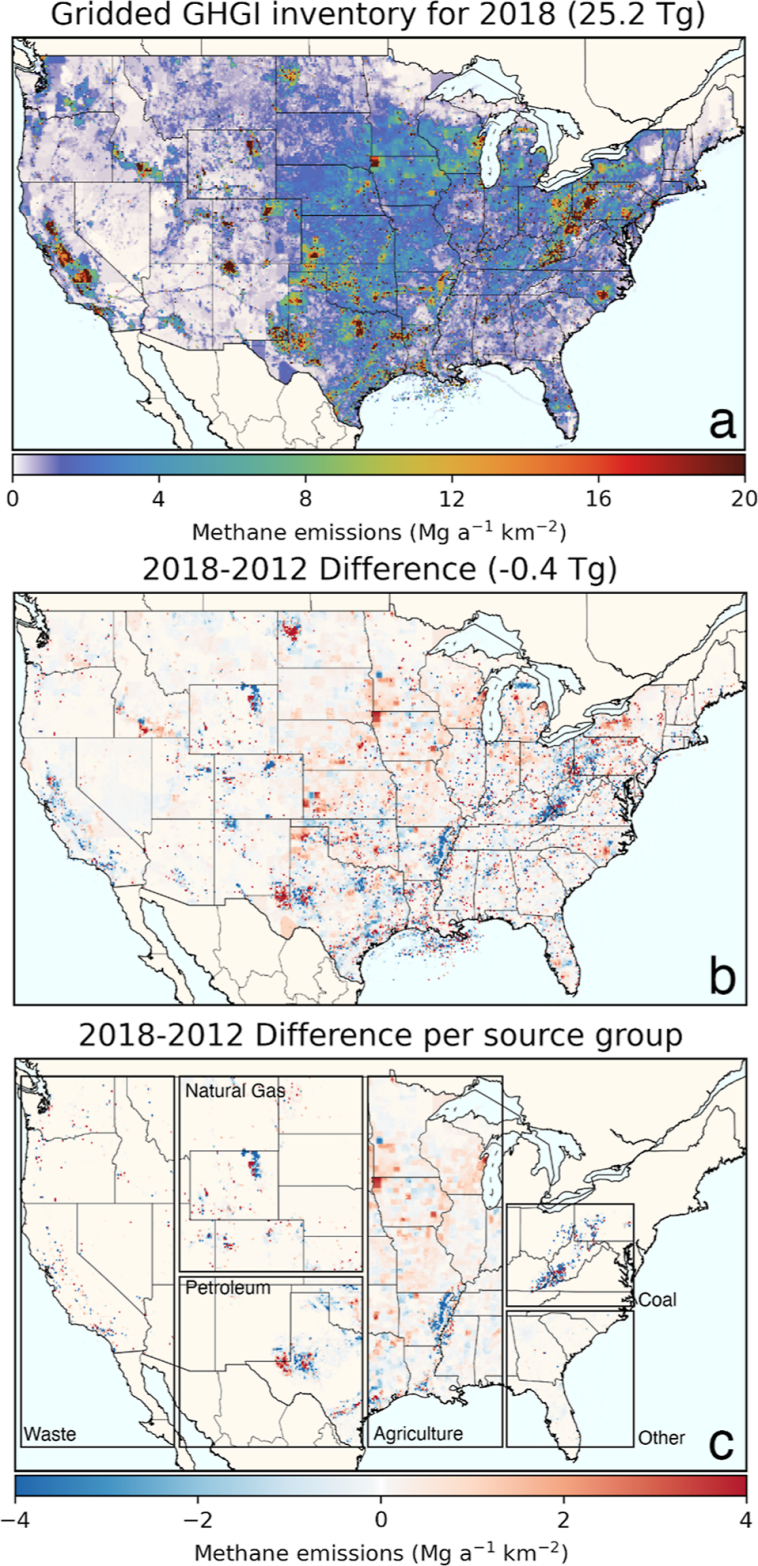
National gridded CONUS methane emissions: (a) absolute 2018 emission fluxes, (b) change in total emission fluxes between 2012 and 2018, and (c) illustration of regional changes in emission fluxes for specific source groups.

CONUS methane emissions from different source sectors exhibit very different spatial patterns. Figure 2 shows gridded 2018 CONUS methane emission fluxes for six aggregate inventory groups (Table 1). Maps of all 26 individual source categories are provided in Figure S1. Agriculture is the largest aggregate source group, with methane emissions widely distributed across the CONUS. These emissions are primarily associated with enteric fermentation and manure management. There are emission hot spots associated with concentrated animal populations, for example, with manure management for dairy cattle (e.g., California, Iowa) and hogs (e.g., North Carolina). Elevated agricultural emissions along the Mississippi River and in northern California are from rice cultivation. In contrast, the spatial patterns in natural gas methane emissions—the second largest source group—are primarily driven by production segment emissions, which are clustered in the large gas-producing basins throughout the Appalachia region, Wyoming, New Mexico, Oklahoma, and Texas. Natural gas exploration and processing emissions tend to follow similar spatial patterns, while transmission and storage segment emissions are more geographically distributed along transmission pipelines and at individual compressor stations and storage sites. Natural gas distribution emissions are concentrated in densely populated areas. Emissions from petroleum systems are centralized in oil-producing basins in North Dakota, Wyoming, Colorado, Kansas, Oklahoma, Texas, and Appalachia, along with emissions at individual refineries. Waste management is the third largest source group, with emissions mainly allocated to large individual point sources, as well as population centers (particularly domestic wastewater treatment). Surface and underground coal mine emissions are largely centralized in southwest Appalachia, with some additional emissions from mines in the Midwest and Alabama as well as emissions from abandoned coal mines that also occur in parts of Colorado and Utah. The spatial patterns of the generally smaller “Other” source group (Table 1) are driven by a combination of point source emissions (e.g., stationary combustion and industrial facilities), those centralized around densely populated areas (e.g., stationary and mobile combustion), and emissions from abandoned oil and gas wells distributed across production regions. Aggregated across all sectors, annual-gridded CONUS emissions (Figure 3a, 25.2 Tg in 2018) are slightly lower than national 2020 GHGI emissions (25.4 Tg in 2018) due to our exclusion of emissions from Alaska, Hawaii, and U.S. territories. Monthly 2018 CONUS emissions vary from 64.8 Gg per day in December to 76.1 Gg per day in June, mainly driven by variability in manure management emissions.

Our data set also reveals temporal changes in spatial patterns of CONUS methane emissions between 2012 and 2018 (Figures 3b,c and S1). While total CONUS emissions decreased by only 2% over these years, there are large regional and sectoral changes. For example, emissions from livestock increased nationally (+7%), mainly due to increased cattle populations and shifts to liquid manure management systems for dairy cattle and swine but also showed decreases in some counties due to reduced animal populations. Emissions along the Mississippi River decreased because of reduced rice production. Similarly, while CONUS methane emissions from natural gas and petroleum exploration and production decreased nationally [exploration emissions due to reduced emission (well) completions^81^ and production emissions due to increased use of low-emitting equipment] despite increased production, regional patterns vary, reflecting local changes in gas well counts and production volumes. For nationally increasing transmission and processing emissions, local changes vary with reported facility-level data, while gas distribution emissions decrease most prominently in the northeast due to a transition from cast iron to less leaky plastic pipelines. CONUS emissions from MSW landfills decreased (−6%) due to increased gas collection despite an increase in landfilled waste, with individual landfills showing large variability based on GHGRP-reported emissions. The largest absolute sectoral decrease comes from coal mining (−0.55 Tg yr^–1^, −19%), associated with decreased coal production and increased methane recovery, which is most clearly visible over Appalachia.

**Figure 4:**
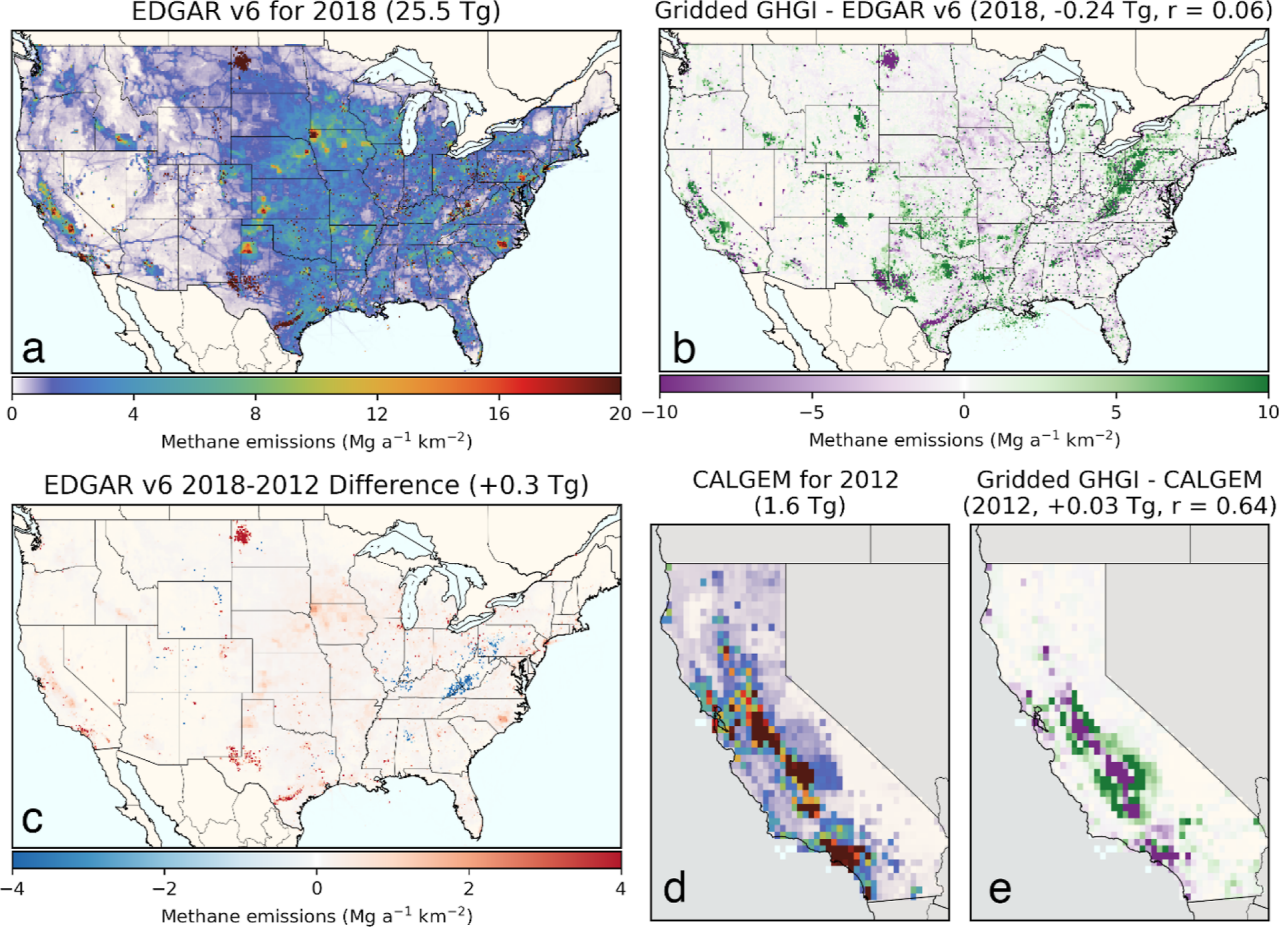
Comparison between the gridded GHGI, EDGAR v6, and CALGEM inventories. (a) Total anthropogenic methane emission fluxes from EDGAR v6 for 2018, (b) gridded GHGI–EDGAR difference for 2018, (c) 2012–2018 trend in EDGAR v6, (d) total livestock, oil and gas, landfill, and wastewater emissions in CALGEM for 2012, and (e) gridded GHGI–CALGEM difference for 2012. (d,e) use the same color scales as (a,b), respectively. Correlation coefficients and totals are calculated over cells with nonzero emissions in both inventories.

As discussed previously, the interpretation of observation-based inversion results will be sensitive to the used prior emission estimates. The EDGAR emissions inventory is often used as a prior estimate for inverse analyses. Figure 4a–c compare our 2018 0.1° × 0.1° gridded GHGI to the global EDGAR v6 inventory^82^ (shown by source group in Figure S2). We use EDGAR v6 because v7^83^ does not contain separate natural gas and petroleum emissions. The comparison shows that differences with the gridded and national GHGI may lead to biases or misinterpretation of inversion results when those analyses draw conclusions about the GHGI. Total CONUS methane emissions are similar between the gridded GHGI and EDGAR (25.2 vs 25.5 Tg, respectively), but Figure 4c reveals large differences in the spatial patterns, mainly driven by differences in the (facility-level) data used by the two products for spatial allocation. For example, in EDGAR, methane emissions from the production of oil and gas are both allocated to spatial patterns that are more representative of oil rather than gas production. As a result, regions with large gas production emissions in the gridded GHGI do not show the same large emissions in EDGAR, while predominantly oil-producing regions have larger hotspots in EDGAR. As a result, the spatial correlation of total anthropogenic methane emissions between the two inventories is close to 0 (*r* = 0.06). In an additional comparison with a different gridded product, we find significant spatial correlation (*r* = 0.64 at 0.2°) in 2012 emissions with the gridded Californian CALGEM inventory.^84^ CALGEM is the most recent version of the only gridded state-specific methane inventory currently available (Figure 4d,e, Table S3). Many of the remaining spatial differences in this comparison are caused by livestock emissions (*r* = 0.47), where additional farm-level information in CALGEM results in more concentrated emissions in California’s Central Valley than those in the gridded GHGI.

In terms of spatial temporal changes, the 2012–2018 trends in EDGAR shown (Figures 4c and S2) are much more spatially uniform than in the gridded GHGI (Figure 3b). This is in part because the underlying data used to spatially allocate EDGAR emissions do not vary as much from year to year. For example, livestock (+7%), oil and gas (+4%), and waste sector (+5%) emissions in EDGAR are estimated to have uniformly increased across the CONUS, while coal emissions uniformly decreased. Not only do some of these national sectoral trends differ in the gridded GHGI (e.g., oil and gas and waste), there is also much larger spatial variability in the trends within each sector, largely from annual changes in reported facility-level and infrastructure data sets that underly the GHGI and are used in the gridded product.

**Figure 5:**
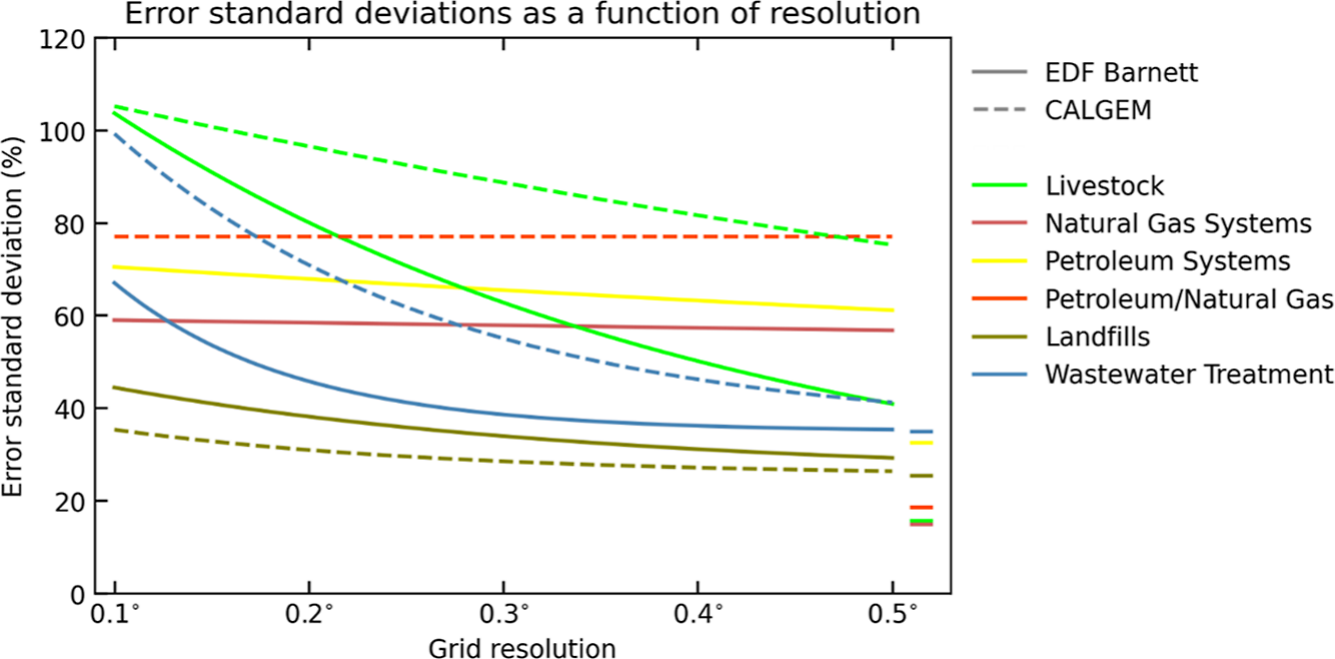
Error standard deviation curves optimized based on comparison of 2012 emissions from the gridded GHGI with the Barnett (solid) and CALGEM (dashed) inventories. Errors are shown as a function of resolution, with solid lines on the right representing the national-level GHGI errors.

Comparison of our emissions to atmospheric observations, for example, in inverse studies, requires characterization of the uncertainty in our gridded estimates. These include uncertainties underlying the development of national estimates (as discussed in the GHGI Report^7^), as well as the gridding methodologies and data sets used here. To assess resolution-dependent uncertainties in our emissions, we compare them to the detailed 2012 bottom-up inventory compiled for the Barnett region in Texas by Lyon et al. (2015)^78^ and adjusted by Zavala-Araiza et al. (2015)^79^ to match atmospheric measurements (Figure S3, and Table S3). Representing the most detailed available regional bottom-up inventory matching atmospheric observations, we take the Barnett inventory as the best available representation of the truth. Figure 5 shows the error curves from eq 1, calibrated based on the difference between the gridded GHGI and Barnett inventories at different spatial resolutions. They illustrate how the spatial allocation error in the gridded estimates is anticipated to decrease at coarser resolutions until reaching the GHGI uncertainty levels at the national scale. The GHGI uncertainties have been updated since the Maasakkers et al. (2016)^29^ analysis of the 2016 GHGI, which is most impactful for petroleum and landfill emissions. For these sectors, national-level errors were reduced enough that we now find a decrease in uncertainty as a function of coarsening resolution, whereas previously, errors were set at the national level for all resolutions.

For livestock emissions, the error quickly decreases at coarser spatial scales as high-resolution misallocation is caused by the lack of subcounty spatial information. For petroleum and natural gas, the uncertainty levels are large (∼60–80%) and relatively flat across resolutions from 0.1° (∼10 km) to 0.5° (∼50 km), suggesting higher basin than national-level uncertainties. For landfills and wastewater treatment, uncertainty levels at 0.5° are similar to the national-level errors and increase only slightly with spatial resolution, partly because these emissions depend on individual facility locations. Compared to Maasakkers et al. (2016),^29^ we find higher correlation of wastewater treatment emissions with the Barnett inventory, even though the comparison is not fully independent as both inventories partly rely on population density for their emission allocation (Figure S3).

Figure 5 also includes error curves estimated based on the comparison to (aggregated) source sectors from the CALGEM inventory, where state-level CALGEM emissions have been scaled to match the gridded GHGI to isolate the spatial allocation errors. These curves generally show similar slopes as the Barnett comparison, except for livestock where the errors decrease slower with coarser resolution due to the large counties in California accentuating the lack of subcounty data. Emissions from oil and gas show limited correlation (*r* = 0.58 at 0.2°), and the mismatch does not quickly decrease when aggregating. By contrast, landfill emissions are strongly spatially correlated (*r* = 0.92 at 0.2°) and fall off similar to the Barnett results. Error parameters for all sources are given in Table S3, including recommendations for those sectors not included in the Barnett analysis.

Our “express extension” emissions data set facilitates preliminary comparisons between more recent observations and GHGI emission estimates but are still based on 2018–2020 source-specific spatial patterns.^33^ CONUS 2018 express emissions (shown per aggregate group in Figure S4) are 6% (1.4 Tg) higher than that in our main product, mainly because of the addition of postmeter emissions (0.44 Tg) and increased natural gas production emissions (0.56 Tg) in the more recent GHGI. While the spatial patterns are held constant for 2018–2020, CONUS emissions in the express data set decrease by 3% (−0.8 Tg) over this time period, mainly driven by decreases in emissions from natural gas production (−0.38 Tg) and coal (−0.48 Tg). Until an updated full-gridded version is released, this express data set serves as the best spatial representation of the 2022 GHGI. These two data sets were developed collaboratively with national inventory compilers and represent the first time series of gridded estimates of reported anthropogenic U.S. methane emissions, enabling improved comparisons between the U.S. GHGI and atmospheric observations.

## Supporting Information Available

Additional supplemental figures and tables are provided in the supplement (PDF)
Both gridded GHGI datasets (2012-2018 gridded GHGI and the 2012-2020 express extension) are available for download at: https://doi.org/10.5281/zenodo.8367082
The methodological code supporting the development and analysis of the gridded GHGI is available for download at: https://github.com/USEPA/GEPA
