# Supplementary material for: A Gridded Inventory of Annual 2012–2018 U.S. Anthropogenic Methane Emissions
Source: Environ Sci Technol. 2023 Oct 19;57(43):16276–88. doi: 10.1021/acs.est.3c05138 (PMC10620993; doi:10.1021/acs.est.3c05138)
Supplement: Supplementary file 1 — es3c05138_si_001.pdf [file es3c05138_si_001.pdf]

# Supplement to:

# A gridded inventory of annual 2012-2018 U.S. anthropogenic methane emissions

Joannes D. Maasakkers[1*], Erin E. McDuffie[2*], Melissa P. Sulprizio[3], Candice Chen[1,3], Maggie Schultz[1], Lily Brunelle[1], Ryan Thrush[1], John Steller[2], Christopher Sherry[2], Daniel J. Jacob[3], Seongeun Jeong[4], Bill Irving[2], and Melissa Weitz[2]

[1]*SRON Netherlands Institute for Space Research, Leiden, Netherlands.*
[2]*Climate Change Division, U.S. Environmental Protection Agency, Washington, DC 20004, USA*
[3]*School of Engineering and Applied Sciences, Harvard University, Cambridge, MA 02138, USA*
[4]*Lawrence Berkeley National Laboratory, Berkeley, CA 94720, USA*
**These authors contributed equally*

## Contents (17 pages):

Tables: S1-S3

Figures: S1-S4

Supplemental references

## Tables

**Table S1.** Gridded CONUS methane emissions (kt $yr^{-1}$) for 2012 and 2018 and the percent change between years, as well as the methodological steps used to grid each source category. Sources are ordered by decreasing 2018 emissions.

| Source (CRF[a] category) | CONUS Emissions (kt) | | | Gridding Methodology[c] | | |
|---|---|---|---|---|---|---|
| | **2012** | **2018** | **Percent Change (%)[b]** | **Step A** | **Step B** | **Step C** |
| **Total** | **25,691** | **25,227** | **-1.8** | **-** | **-** | **-** |
| **Agriculture** | 9556 | 10106 | +5.8 | - | - | - |
| *Enteric fermentation (3A)* | 6658 | 7091 | +6.5 | X | X | X |
| *Manure management (3B)** | 2277 | 2465 | +8.3 | X | X | X |
| *Rice cultivation (3C)** | 606 | 533 | -12.0 | X | X | X |
| *Field burning of agricultural residues (3F)** | 14 | 16 | +14.3 | X | - | X |
| **Natural Gas Systems (1B2b)** | **5650** | **5590** | **-1.1** | **-** | **-** | **-** |
| *Production** | 3489 | 3237 | -7.2 | X[d] | - | X |
| *Transmission & Storage* | 1165 | 1352 | +16.1 | - | - | X |
| *Processing* | 398 | 486 | +22.1 | - | - | X |
| *Distribution* | 499 | 472 | -5.4 | X | - | X |
| *Exploration** | 100 | 44 | -56.0 | - | - | X |
| **Waste** | **5204** | **4993** | **-4.1** | **-** | **-** | **-** |
| *Municipal Solid Waste (MSW) landfills (5A1)* | 3961 | 3734 | -5.7 | - | - | X |
| *Industrial landfills (5A1)* | 587 | 594 | +1.2 | X | - | X |
| *Domestic wastewater treatment and discharge (5D)* | 358 | 333 | -7.0 | - | - | X |
| *Industrial wastewater treatment and discharge (5D)* | 221 | 234 | +5.9 | - | - | X |
| *Composting (5B1)* | 77 | 98 | +27.3 | X | - | X |
| **Coal Mines (1B1a)** | **2902** | **2352** | **-19.0** | **-** | **-** | **-** |
| *Underground coal mining* | 2158 | 1766 | -18.2 | X | - | X |
| *Surface coal mining* | 495 | 339 | -31.5 | X | - | X |
| *Abandoned underground coal mines* | 249 | 247 | -0.8 | - | - | X |
| **Petroleum Systems (1B2a)** | **1601** | **1426** | **-10.9** | **-** | **-** | **-** |
| *Production** | 1262 | 1373 | +8.8 | - | - | X |
| *Refining** | 27 | 30 | +11.1 | - | - | X |
| *Exploration** | 306 | 15 | -95.1 | - | - | X |
| *Transport** | 6 | 8 | +33.3 | - | - | X |
| **Other** | **782** | **758** | **-3.1** | **-** | **-** | **-** |
| *Stationary combustion (1A) ** | 298 | 340 | +14.1 | X[d] | X[d] | X |
| *Abandoned Oil and Gas wells (1B2a & 1B2b)* | 281 | 281 | 0 | X | - | X |
| *Mobile Combustion (1A)* | 200 | 124 | -38.0 | X[d] | - | X |
| *Petrochemical Production (2B8)* | 3 | 12 | +300 | - | - | X |
| *Ferroalloy production (2C2)* | 1 | 1 | 0 | - | - | x |

[a] Categories reported in UNFCCC Common Reporting Format tables
[b] Calculated as 100* (2018 emissions -2012 emissions) / 2012 emissions
[c] 'X' indicates which gridding steps (see Figure 1 for reference) were used to spatially allocate national emissions. For example, sources with an 'X' for only Step C indicates that national emissions are directly allocated to the grid level. A source category with an 'X' in Steps A and C indicate that national emissions were first allocated to the state-level and then from the state to the grid-level. The use of specific gridding steps reflects the availability of spatially explicit activity data for each source.
[d] Only a subset of sources in this category are allocated to this level.
* Source sectors that include annual gridded emissions and monthly scale factors

**Table S2.** Source-specific methane emission uncertainties. Includes national error estimates from the 2020 GHGI[1], as well as gridded uncertainty estimates. The errors for the first five source sectors were estimated based on comparison with the Barnett inventory[2, 3]. For other sectors, we recommend which (evaluated) source sector to base resolution-dependent error on.

| **Errors optimized based on comparison to the Barnett inventory** | | | |
|---|---|---|---|
| **Source (CRF[a] category)** | **GHGI national uncertainty[b]** | **Additional 0.1° error** | **Error decay coefficient** |
| Livestock (3A+3B) | 15.5%[c] | 88% | 3.12 |
| Natural Gas Systems (1B2b) | 14.5% | 44% | 0.13 |
| Landfills (5A1) | 25.5%[c] | 19% | 4.02 |
| Wastewater treatment and discharge (5D) | 35%[c] | 32% | 10.86 |
| Petroleum Systems (1B2a) | 32.5% | 38% | 0.71 |

| **Source categories for which errors were not (individually) optimized** | | |
|---|---|---|
| **Source (CRF[a] category)** | **GHGI national uncertainty[b]** | **Recommended source sector to base resolution-dependent error on** |
| Enteric fermentation (3A) | 14.5% | Livestock |
| Manure management (3B) | 19% | Livestock |
| Rice cultivation (3C) | 46.5% | Livestock |
| Field burning of agricultural residues (3F) | 16% | Livestock |
| Municipal Solid Waste (MSW) landfills (5A1) | 25% | Landfills |
| Industrial landfills (5A1) | 28% | Landfills |
| Domestic wastewater treatment and discharge (5D) | 25% | Wastewater |
| Industrial wastewater treatment and discharge (5D) | 49% | Wastewater |
| Composting (5B1) | 50% | Wastewater |
| Underground coal mining | 14.5% | Landfills |
| Surface coal mining | 14.5% | Landfills |
| Abandoned underground coal mines | 17.5% | Wastewater |
| Stationary combustion (1A) | 82.5% | Wastewater |
| Abandoned Oil and Gas wells (1B2a & 1B2b) | 151% | Petroleum |
| Mobile Combustion (1A) | 17.5% | Wastewater |
| Petrochemical Production (2B8) | 51.5% | Landfills |
| Ferroalloy production (2C2) | 12% | Landfills |

[a] Categories reported in UNFCCC Common Reporting Format tables
[b] Average of the confidence interval
[c] Calculated as the weighted average of the subsector uncertainties.

**Table S3.** Regional methane emissions totals (kt $yr^{-1}$) for 2012 from multiple gridded inventories. Sources are ordered by decreasing 2012 emissions. CALGEM comparisons are done at 0.2° resolution as the inventories are on grids offset by 0.05°.

| **California** | | | |
|---|---|---|---|
| **Source group** | **Gridded GHGI** | **CALGEM[4]** | ***r*** |
| Livestock | 843 | 899 | 0.47 |
| Landfills | 434 | 337 | 0.92 |
| Petroleum and Natural Gas systems | 234 | 284 | 0.58 |
| Wastewater | 41 | 67 | 0.66 |
| **Total** | **1552** | **1587** | **0.64** |

| **Barnett** | | | |
|---|---|---|---|
| **Source group** | **Gridded GHGI** | **EDF Barnett inventory[2, 3]** | ***r*** |
| Natural gas systems | 279 | 472 | 0.81 |
| Production (and exploration) | 237 | 396 | 0.86 |
| Processing | 19 | 65 | 0.394 |
| Transmission and storage | 13 | 2 | 0.18 |
| Distribution | 9 | 9 | 0.87 |
| Livestock | 114 | 101 | 0.36 |
| Landfills | 98 | 99 | 0.65 |
| Petroleum systems | 43 | 39 | 0.51 |
| Wastewater treatment | 7 | 7 | 0.67 |
| **Total** | **541** | **718** | **0.67** |

# Figures

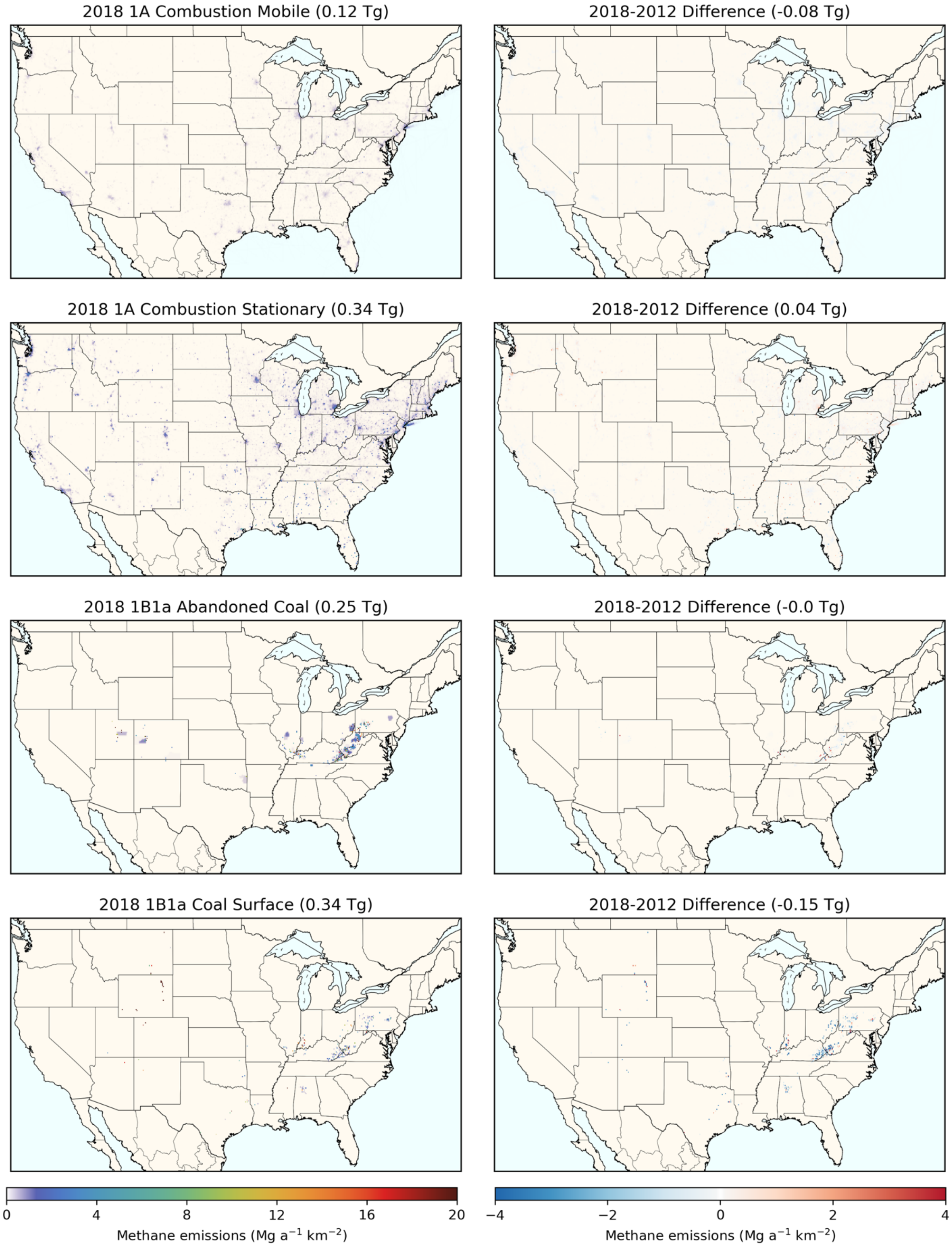
2018 1A Combustion Mobile (0.12 Tg)
2018-2012 Difference (-0.08 Tg)
2018 1A Combustion Stationary (0.34 Tg)
2018-2012 Difference (0.04 Tg)
2018 1B1a Abandoned Coal (0.25 Tg)
2018-2012 Difference (-0.0 Tg)
2018 1B1a Coal Surface (0.34 Tg)
2018-2012 Difference (-0.15 Tg)
0 4 8 12 16 20
Methane emissions (Mg a$^{-1}$ km$^{-2}$)
−4 −2 0 2 4
Methane emissions (Mg a$^{-1}$ km$^{-2}$)

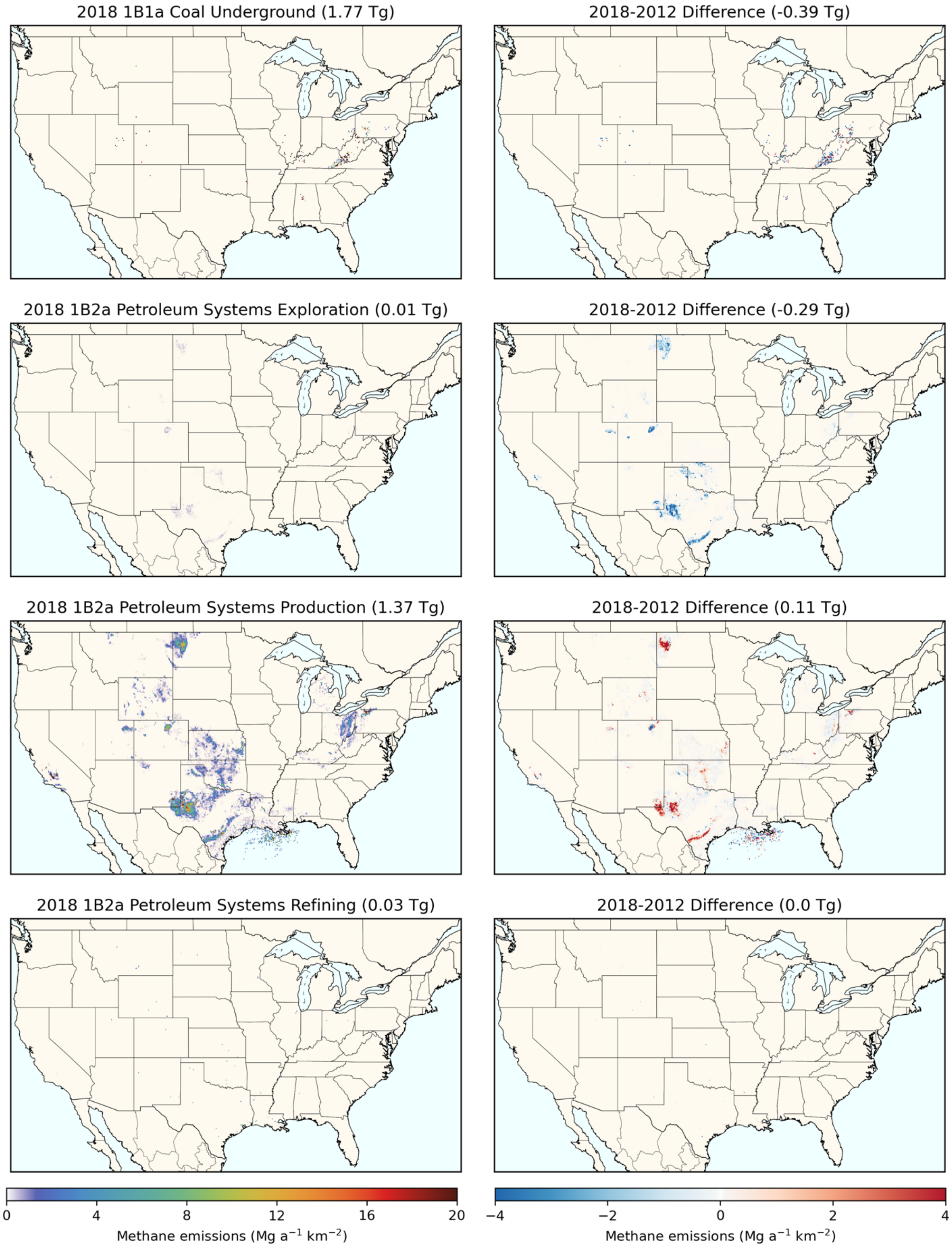
2018 1B1a Coal Underground (1.77 Tg)
2018-2012 Difference (-0.39 Tg)
2018 1B2a Petroleum Systems Exploration (0.01 Tg)
2018-2012 Difference (-0.29 Tg)
2018 1B2a Petroleum Systems Production (1.37 Tg)
2018-2012 Difference (0.11 Tg)
2018 1B2a Petroleum Systems Refining (0.03 Tg)
2018-2012 Difference (0.0 Tg)
0 4 8 12 16 20
Methane emissions (Mg a$^{-1}$ km$^{-2}$)
−4 −2 0 2 4
Methane emissions (Mg a$^{-1}$ km$^{-2}$)

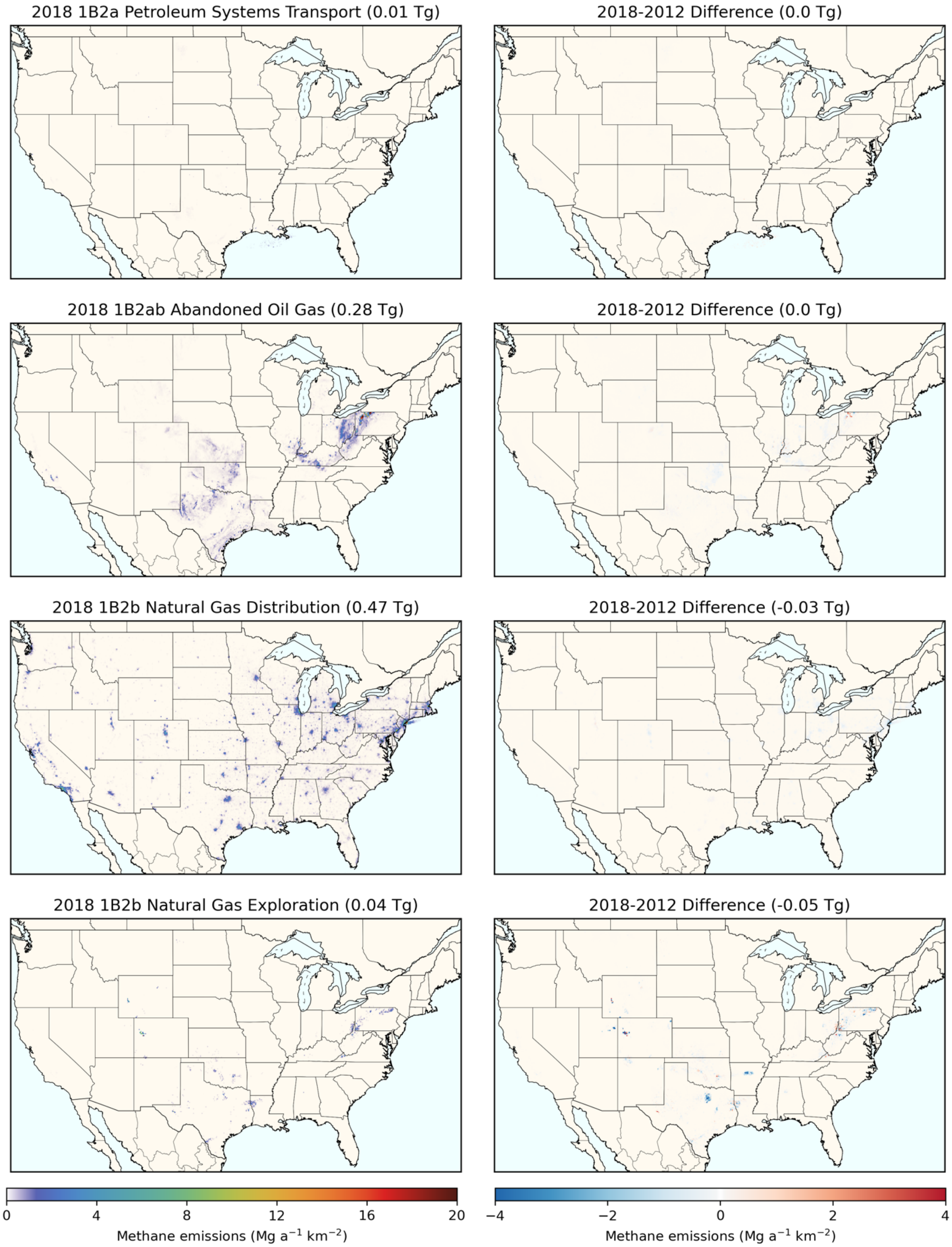
2018 1B2a Petroleum Systems Transport (0.01 Tg)
2018-2012 Difference (0.0 Tg)
2018 1B2ab Abandoned Oil Gas (0.28 Tg)
2018-2012 Difference (0.0 Tg)
2018 1B2b Natural Gas Distribution (0.47 Tg)
2018-2012 Difference (-0.03 Tg)
2018 1B2b Natural Gas Exploration (0.04 Tg)
2018-2012 Difference (-0.05 Tg)
0
4
8
12
16
20
Methane emissions (Mg a$^{-1}$ km$^{-2}$)
−4
−2
0
2
4
Methane emissions (Mg a$^{-1}$ km$^{-2}$)

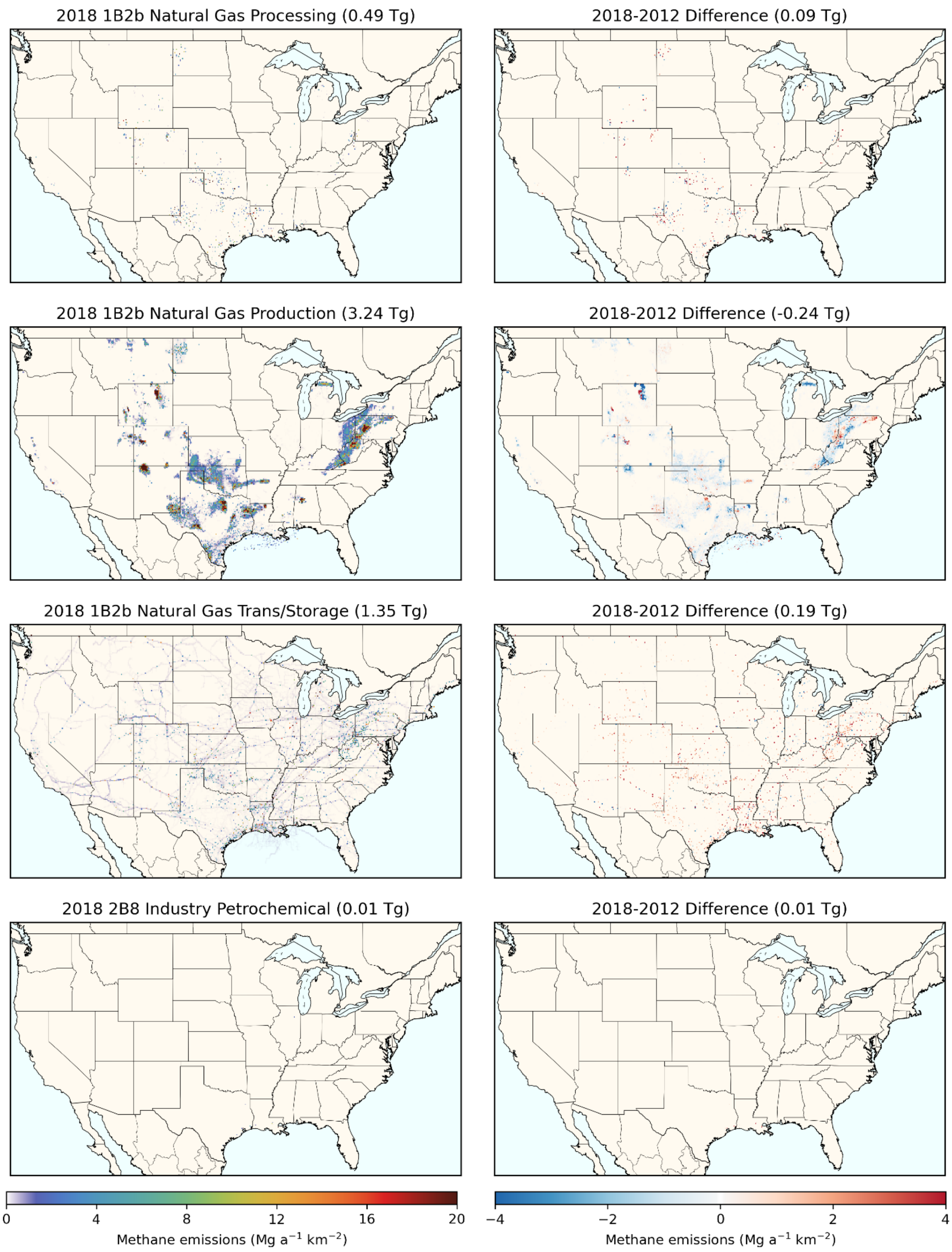
2018 1B2b Natural Gas Processing (0.49 Tg)
2018-2012 Difference (0.09 Tg)
2018 1B2b Natural Gas Production (3.24 Tg)
2018-2012 Difference (-0.24 Tg)
2018 1B2b Natural Gas Trans/Storage (1.35 Tg)
2018-2012 Difference (0.19 Tg)
2018 2B8 Industry Petrochemical (0.01 Tg)
2018-2012 Difference (0.01 Tg)
0
4
8
12
16
20
Methane emissions (Mg a−1 km−2)
−4
−2
0
2
4
Methane emissions (Mg a−1 km−2)

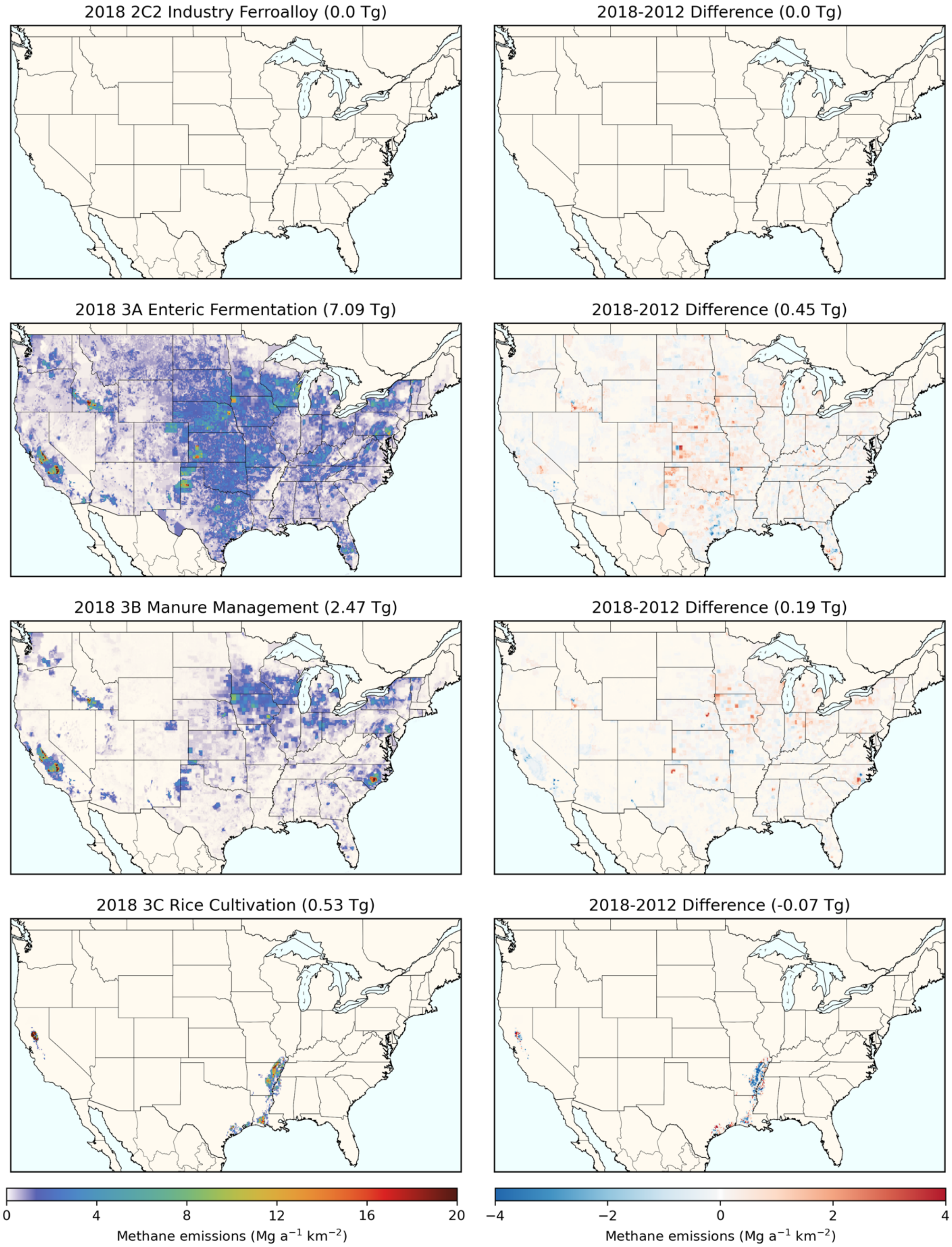
2018 2C2 Industry Ferroalloy (0.0 Tg)
2018-2012 Difference (0.0 Tg)
2018 3A Enteric Fermentation (7.09 Tg)
2018-2012 Difference (0.45 Tg)
2018 3B Manure Management (2.47 Tg)
2018-2012 Difference (0.19 Tg)
2018 3C Rice Cultivation (0.53 Tg)
2018-2012 Difference (-0.07 Tg)
0 4 8 12 16 20
Methane emissions (Mg a$^{-1}$ km$^{-2}$)
−4 −2 0 2 4
Methane emissions (Mg a$^{-1}$ km$^{-2}$)

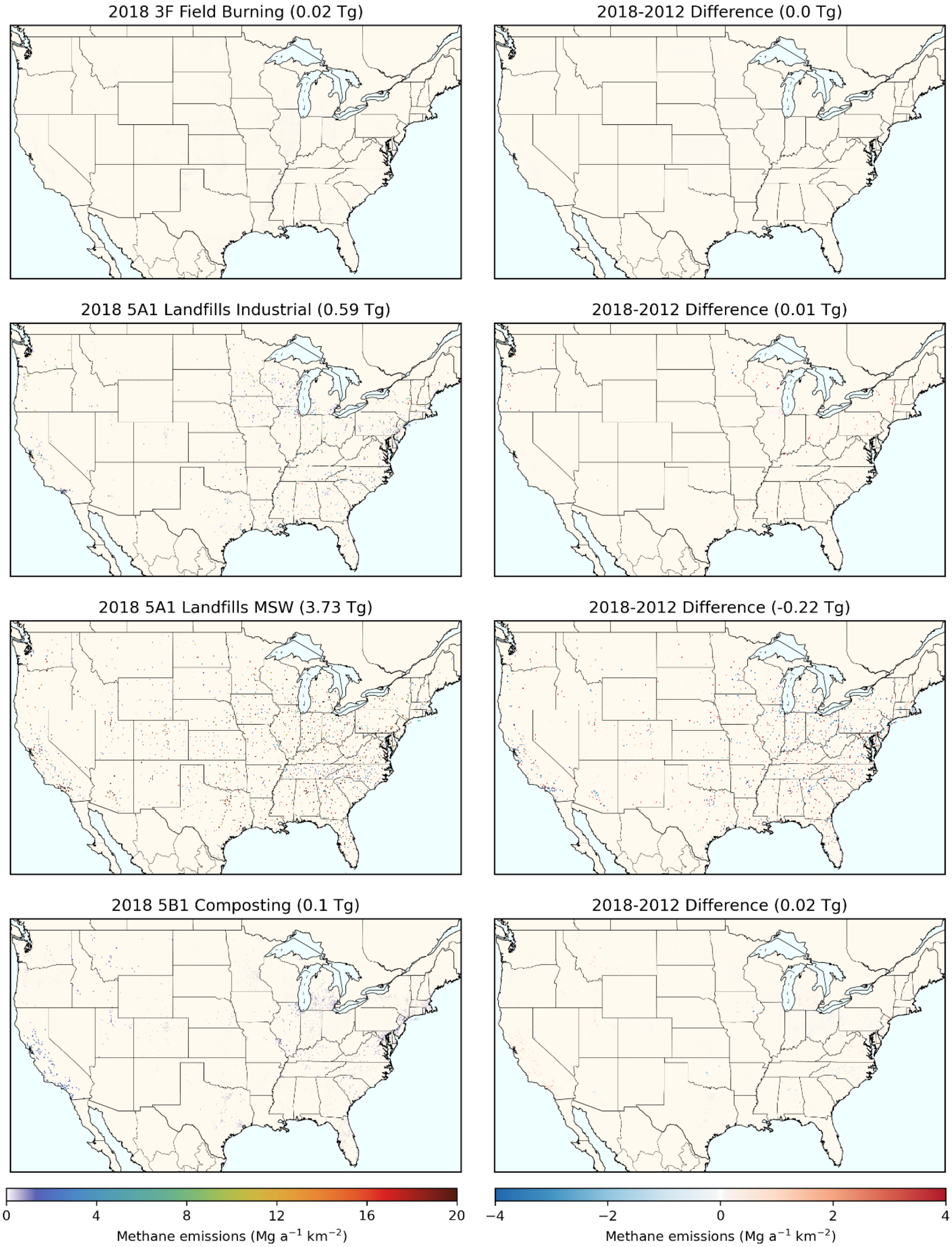
2018 3F Field Burning (0.02 Tg)
2018-2012 Difference (0.0 Tg)
2018 5A1 Landfills Industrial (0.59 Tg)
2018-2012 Difference (0.01 Tg)
2018 5A1 Landfills MSW (3.73 Tg)
2018-2012 Difference (-0.22 Tg)
2018 5B1 Composting (0.1 Tg)
2018-2012 Difference (0.02 Tg)
0 4 8 12 16 20
Methane emissions (Mg a$^{-1}$ km$^{-2}$)
−4 −2 0 2 4
Methane emissions (Mg a$^{-1}$ km$^{-2}$)

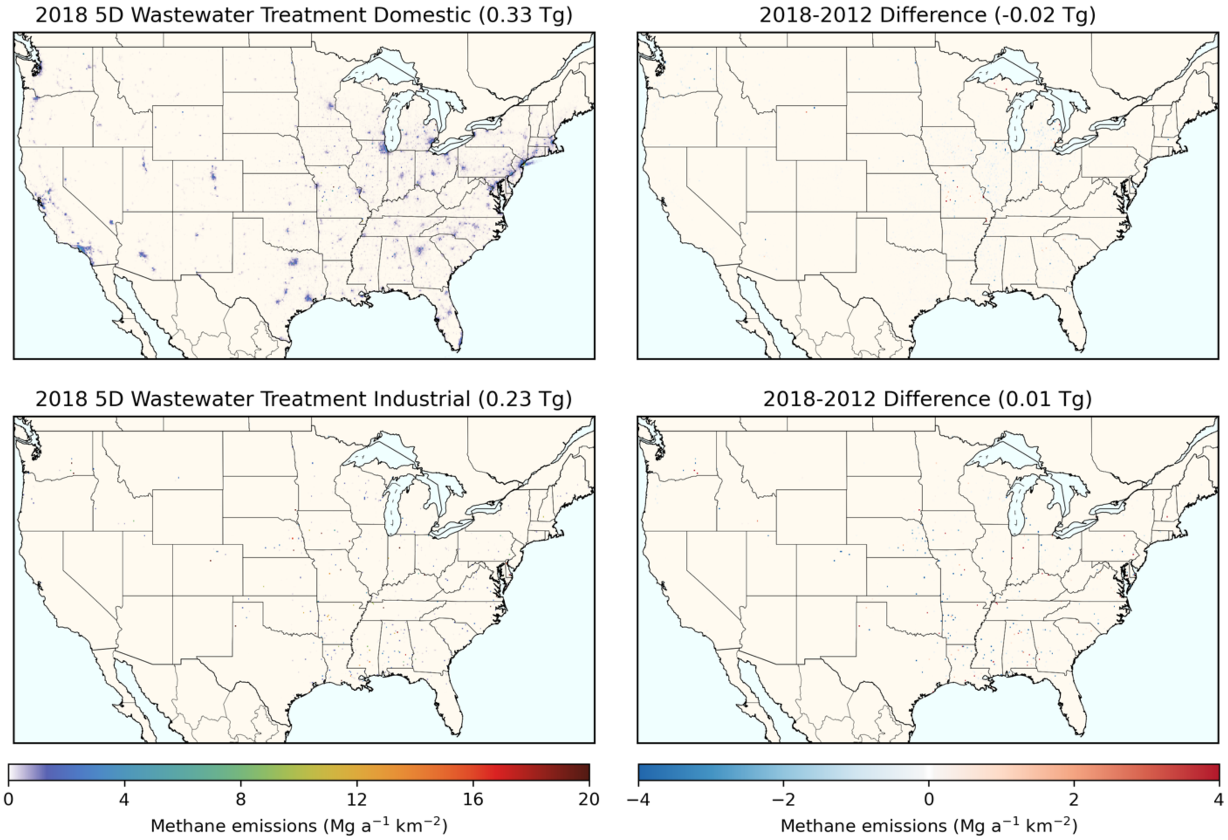


**Figure S1.** Gridded emissions for 26 aggregate inventory source categories. Left column) absolute emission fluxes in 2018. Right column) change in emission fluxes between 2012 and 2018 (2018-2012). Emissions are in megagrams (million metric tons) per year per squared kilometer.

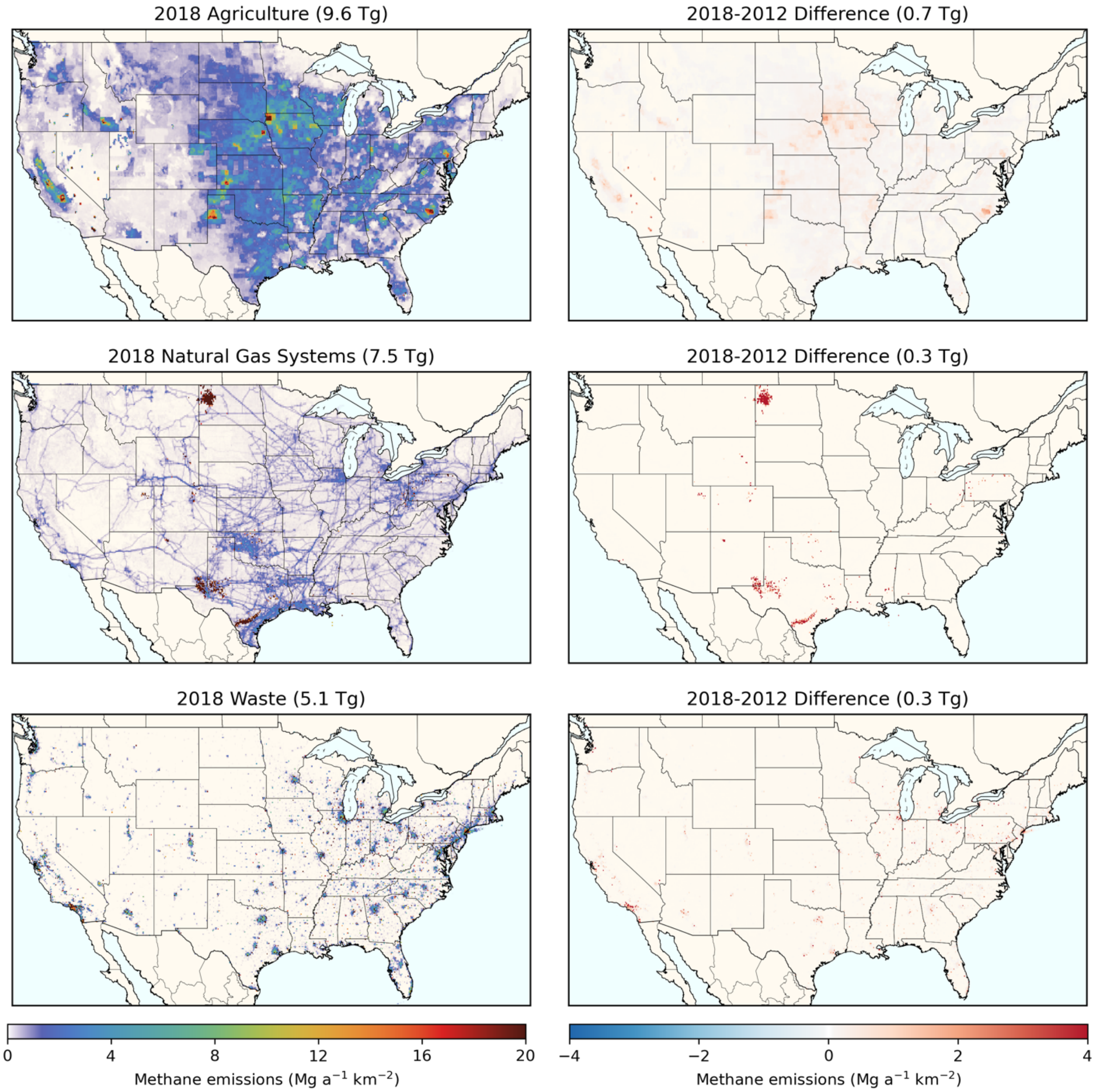
2018 Agriculture (9.6 Tg)
2018-2012 Difference (0.7 Tg)
2018 Natural Gas Systems (7.5 Tg)
2018-2012 Difference (0.3 Tg)
2018 Waste (5.1 Tg)
2018-2012 Difference (0.3 Tg)
0
4
8
12
16
20
Methane emissions (Mg a$^{-1}$ km$^{-2}$)
−4
−2
0
2
4
Methane emissions (Mg a$^{-1}$ km$^{-2}$)

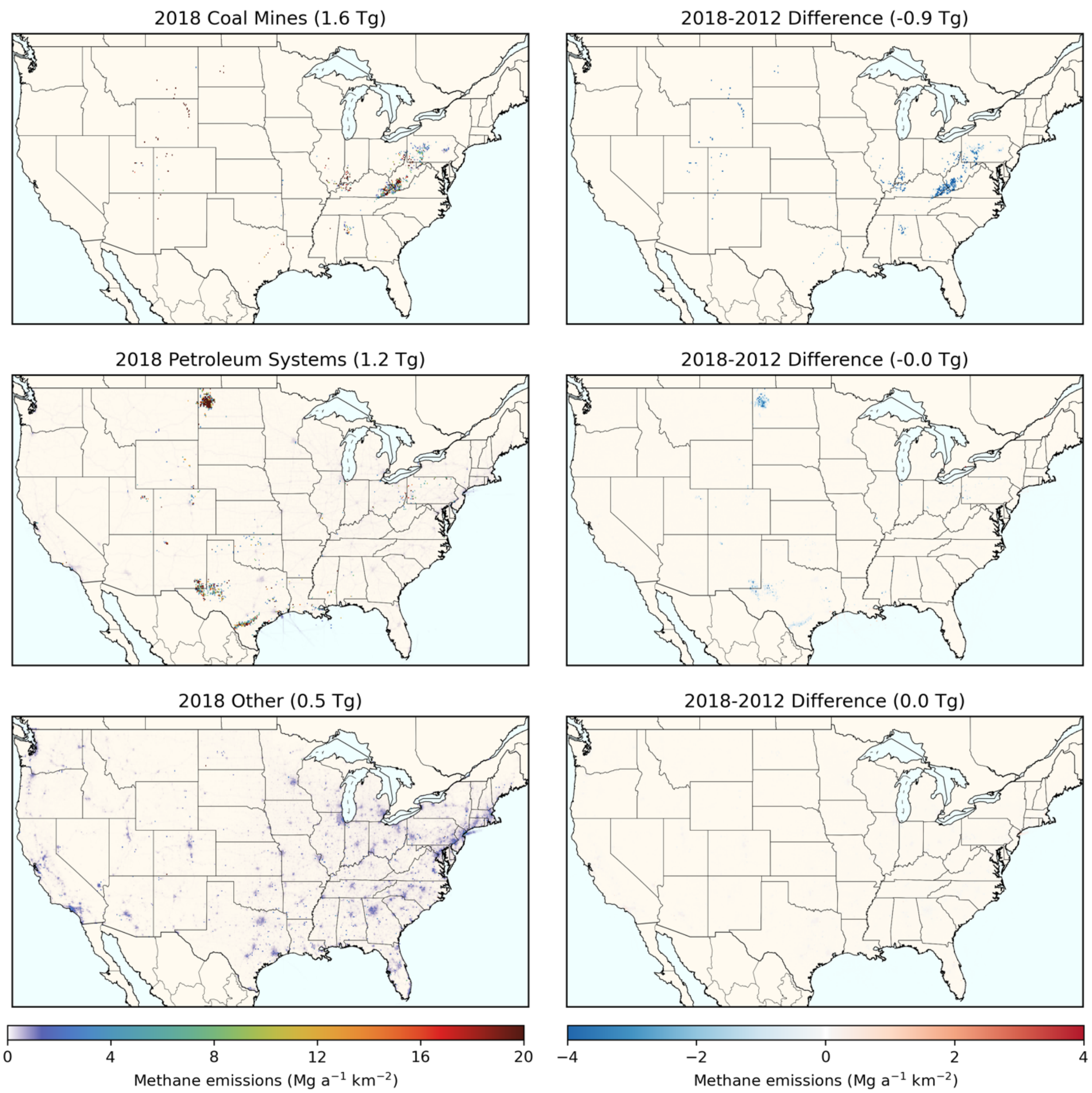


**Figure S2.** Gridded EDGAR v6 emissions for the six aggregate source groups from Table 1 and Figure 1. Left column) absolute emission fluxes in 2018. Right column) change in emission fluxes between 2012 and 2018 (2018-2012). Emissions are in megagrams (million metric tons) per year per squared kilometer.

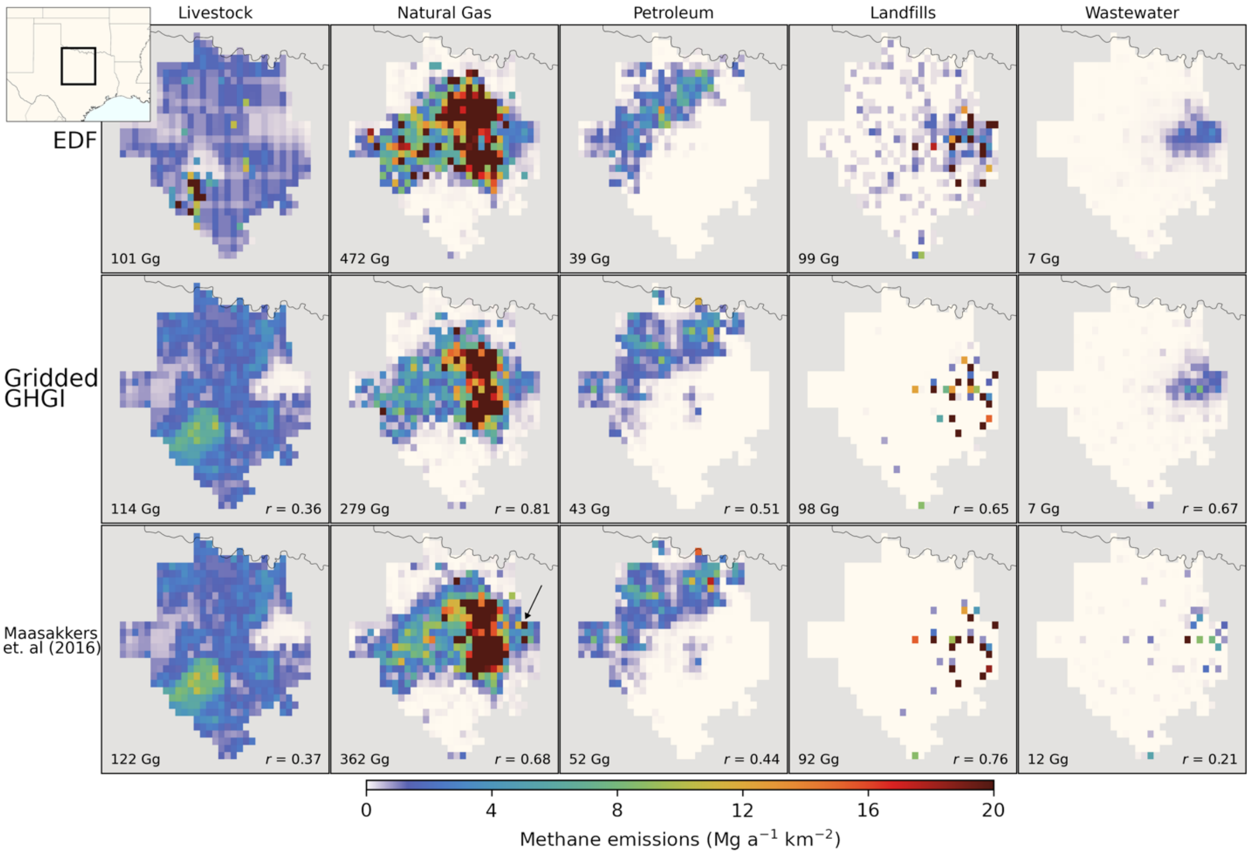


**Figure S3.** Comparison between the 2012 regional Barnett inventory[2, 3] (top row, originally produced at 4×4 $km^2$ and regridded to 0.1°×0.1° here), our gridded GHGI (middle row), and Maasakkers et al. (2016)[5] (bottom row) inventories over central Texas. Panels show total sectoral emissions over the spatial extent of the Barnett inventory and spatial correlation coefficients with the Barnett inventory. The city of Dallas, where natural gas processing emissions were erroneously allocated to company headquarters in Maasakkers et al. (2016) is marked with a black arrow in the Natural Gas panel.

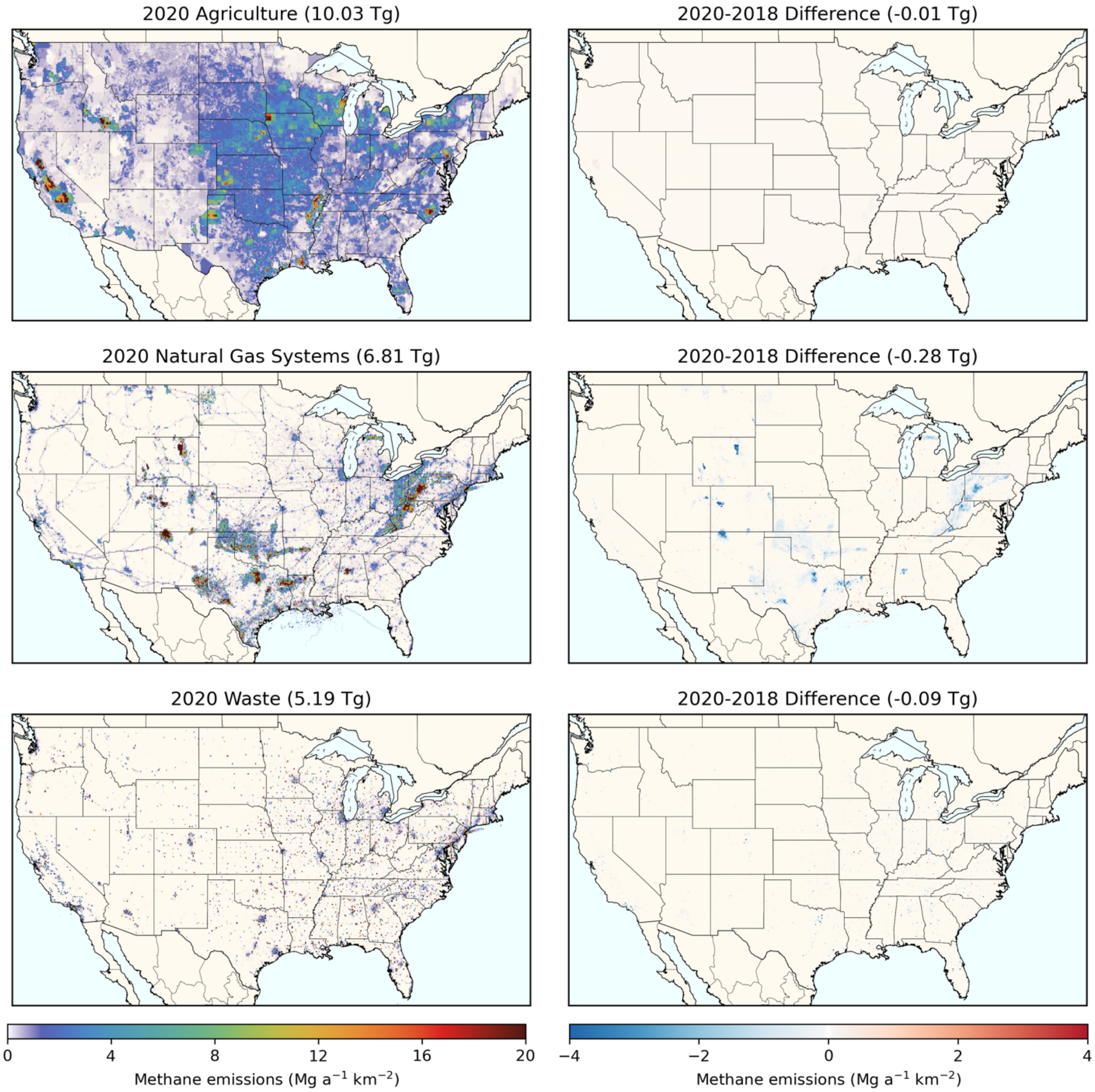
2020 Agriculture (10.03 Tg)
2020-2018 Difference (-0.01 Tg)
2020 Natural Gas Systems (6.81 Tg)
2020-2018 Difference (-0.28 Tg)
2020 Waste (5.19 Tg)
2020-2018 Difference (-0.09 Tg)
0
4
8
12
16
20
Methane emissions (Mg a$^{-1}$ km$^{-2}$)
−4
−2
0
2
4
Methane emissions (Mg a$^{-1}$ km$^{-2}$)

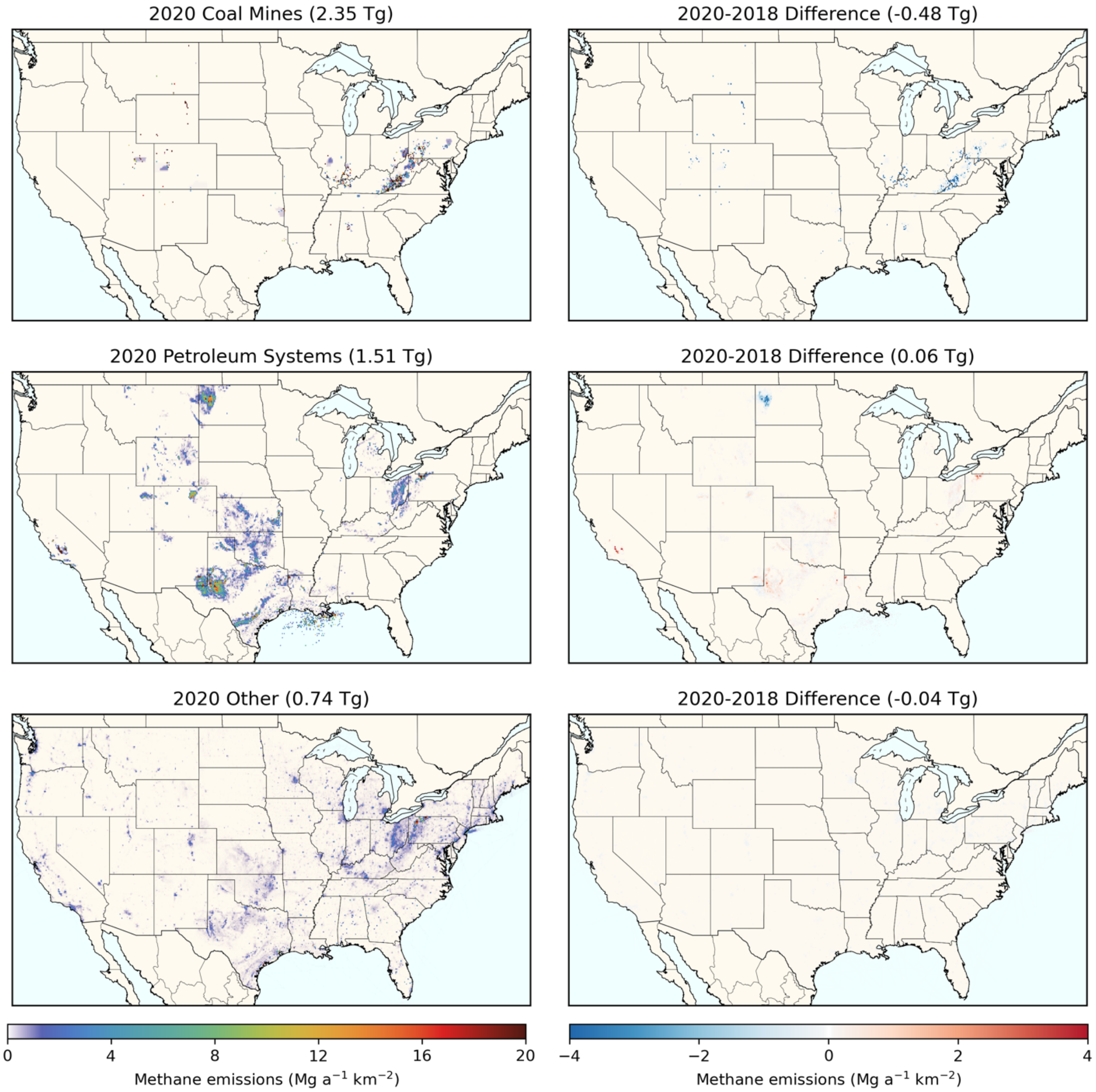


**Figure S4.** Express extension emissions data for the six aggregate source groups from Table 1 and Figure 1. Left column) absolute emission fluxes in 2020. Right column) change in emission fluxes between 2018 and 2020 (2020-2018). Emissions are in megagrams (million metric tons) per year per squared kilometer.

**References**

1. U.S. Environmental Protection Agency (EPA) *Inventory of U.S. Greenhouse Gas Emissions and Sinks: 1990-2018*; USEPA: USA, 2020.

2. Lyon, D. R.; Zavala-Araiza, D.; Alvarez, R. A.; Harriss, R.; Palacios, V.; Lan, X.; Talbot, R.; Lavoie, T.; Shepson, P.; Yacovitch, T. I.; Herndon, S. C.; Marchese, A. J.; Zimmerle, D.; Robinson, A. L.; Hamburg, S. P., Constructing a Spatially Resolved Methane Emission Inventory for the Barnett Shale Region. *Environmental Science & Technology* **2015,** *49* (13), 8147-8157.

3. Zavala-Araiza, D.; Lyon, D. R.; Alvarez, R. A.; Davis, K. J.; Harriss, R.; Herndon, S. C.; Karion, A.; Kort, E. A.; Lamb, B. K.; Lan, X.; Marchese, A. J.; Pacala, S. W.; Robinson, A. L.; Shepson, P. B.; Sweeney, C.; Talbot, R.; Townsend-Small, A.; Yacovitch, T. I.; Zimmerle, D. J.; Hamburg, S. P., Reconciling divergent estimates of oil and gas methane emissions. *Proceedings of the National Academy of Sciences of the United States of America* **2015,** *112* (51), 15597-15602.

4. Jeong, S.; Newman, S.; Zhang, J.; Andrews, A. E.; Bianco, L.; Bagley, J.; Cui, X.; Graven, H.; Kim, J.; Salameh, P.; LaFranchi, B. W.; Priest, C.; Campos-Pineda, M.; Novakovskaia, E.; Sloop, C. D.; Michelsen, H. A.; Bambha, R. P.; Weiss, R. F.; Keeling, R.; Fischer, M. L., Estimating methane emissions in California's urban and rural regions using multitower observations. *Journal of Geophysical Research: Atmospheres* **2016,** *121* (21).

5. Maasakkers, J. D.; Jacob, D. J.; Sulprizio, M. P.; Turner, A. J.; Weitz, M.; Wirth, T.; Hight, C.; DeFigueiredo, M.; Desai, M.; Schmeltz, R.; Hockstad, L.; Bloom, A. A.; Bowman, K. W.; Jeong, S.; Fischer, M. L., Gridded National Inventory of U.S. Methane Emissions. *Environmental Science & Technology* **2016,** *50* (23), 13123-13133.
